# Circuits Regulating Pleasure and Happiness: The Evolution of the Amygdalar-Hippocampal-Habenular Connectivity in Vertebrates

**DOI:** 10.3389/fnins.2016.00539

**Published:** 2016-11-22

**Authors:** Anton J. M. Loonen, Svetlana A. Ivanova

**Affiliations:** ^1^Department of Pharmacy, University of GroningenGroningen, Netherlands; ^2^GGZ Westelijk Noord-Brabant (GGZ-WNB)Halsteren, Netherlands; ^3^Mental Health Research Institute, Tomsk National Research Medical Center of the Russian Academy of SciencesTomsk, Russia; ^4^Department of Ecology and Basic Safety, National Research Tomsk Polytechnic UniversityTomsk, Russia

**Keywords:** cerebral cortex, amygdala, hippocampus, habenula, evolution, reward, stress

## Abstract

Appetitive-searching (reward-seeking) and distress-avoiding (misery-fleeing) behavior are essential for all free moving animals to stay alive and to have offspring. Therefore, even the oldest ocean-dwelling animal creatures, living about 560 million years ago and human ancestors, must have been capable of generating these behaviors. The current article describes the evolution of the forebrain with special reference to the development of the misery-fleeing system. Although, the earliest vertebrate ancestor already possessed a dorsal pallium, which corresponds to the human neocortex, the structure and function of the neocortex was acquired quite recently within the mammalian evolutionary line. Up to, and including, amphibians, the dorsal pallium can be considered to be an extension of the medial pallium, which later develops into the hippocampus. The ventral and lateral pallium largely go up into the corticoid part of the amygdala. The striatopallidum of these early vertebrates becomes extended amygdala, consisting of centromedial amygdala (striatum) connected with the bed nucleus of the stria terminalis (pallidum). This amygdaloid system gives output to hypothalamus and brainstem, but also a connection with the cerebral cortex exists, which in part was created after the development of the more recent cerebral neocortex. Apart from bidirectional connectivity with the hippocampal complex, this route can also be considered to be an output channel as the fornix connects the hippocampus with the medial septum, which is the most important input structure of the medial habenula. The medial habenula regulates the activity of midbrain structures adjusting the intensity of the misery-fleeing response. Within the bed nucleus of the stria terminalis the human homolog of the ancient lateral habenula-projecting globus pallidus may exist; this structure is important for the evaluation of efficacy of the reward-seeking response. The described organization offers a framework for the regulation of the stress response, including the medial habenula and the subgenual cingulate cortex, in which dysfunction may explain the major symptoms of mood and anxiety disorders.

## Introduction

Behavior can be considered as a mechanism whereby the brain manages input in order to create a specific output which enables the organism to adapt to the ever changing circumstances within its biosphere. In humans, input from the senses is primarily translated within the cerebral cortex into a specific behavioral output. Sensory information is processed within the posterior cerebral cortex in a stepwise fashion (Loonen, 2013). Specific information is integrated with other sensory information and transmitted from the primary sensory cortex to the secondary sensory cortex, then to the association cortex, and so on. Within the anterior cerebral cortex, a similar, but now diverging, flow of information occurs which leads to the activation of specific brain regions, for example, the motor cortex. Apart from this stepwise processing, other fibers that run in parallel connect to more distant regions. Every neural connection is capable of learning, due to the characteristics of glutamatergic transmission, which can increase or decrease the sensitivity of connecting synapses by inducing long term potentiation (LTP) or long term depression (LTD). Therefore, the cortex can “learn” to transmit specific sensory information to a specific output unit via a “preferred” cortical tract. Accordingly, the cerebral cortex learns to interpret sensory information and produce a specific behavioral response (Loonen, 2013). This cortical processing can be considered the first level of regulating behavior.

**Figure 1:**
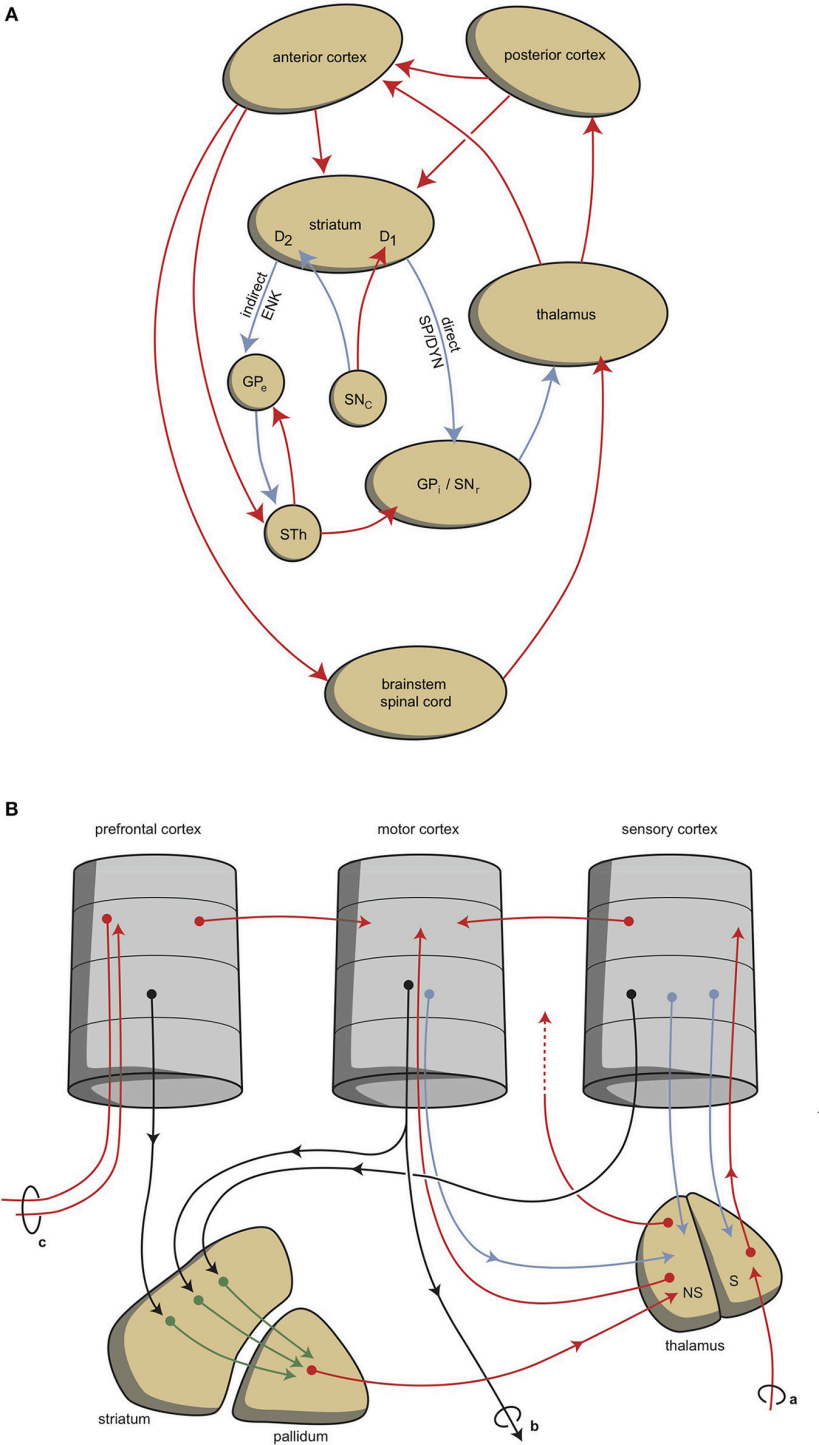
**Scheme representing the organization of human extrapyramidal system. (A)** Direct and indirect pathways lead to the activation (red) or inhibition (blue) of the anterior cortical endpoint. D1, medium spiny neurons carrying dopamine D1 receptors; D2, medium spiny neurons carrying dopamine D2 receptors; DYN, dynorphin; ENK, enkephalin; GPe, globus pallidus externa; GPi, globus pallidus interna; SP, substance P; STh, subthalamic nucleus. **(B)** Convergent pathways via the basal ganglia correct the serially connected intracortical connections. a, sensory input; b, motor output; c, to contralateral cortex; NS, non-specific part of the thalamus; S, specific part of the thalamus. Red, black, green, and blue arrows = neurochemically undetermined.

Although this process is expedient, it can be highly sensitive to dysregulation, both in routine functions and learning. Therefore, a parallel circuit has evolved, which includes subcortical structures (Figure 1). All processing units in the cerebral cortex also send information to the basal ganglia (Heimer, 2003). The route through the basal ganglia and thalamus targets the corresponding processing units in the anterior cortex (Loonen and Ivanova, 2013). This parallel circuit has stimulatory (direct) and inhibitory (indirect) pathways, and its glutamatergic synapses can also induce LTP and LTD (Figure 1). Therefore, this parallel route through the basal ganglia enables the brain to correct serially transmitted information when it arrives at the “final” destination. Moreover, the connection through the basal ganglia is convergent (Figure 1; Alexander et al., 1986; Groenewegen, 2003; Loonen and Ivanova, 2013). Hence, the processing units in the posterior and anterior cortices and their outputs converge within this subcortical circuit, and have the same frontal cortical output unit as the intracortical pathways. Again, the “learning” ability of glutamatergic synapses within this framework make it possible to process a constantly varying input and produce very complex, sophisticated output patterns in a reproducible, precise fashion.

This organization of connections represents the second level of regulation and is well-known as the extrapyramidal system, which adjusts cognition and movements (Groenewegen, 2003). In our mental functions regulatory model, we suggest that a similar organization can be distinguished within the limbic cortex, although here the structure is more complex and less modular due to the ancient origins of these structures (Loonen and Ivanova, 2015, 2016a; Loonen et al., submitted). To simplify, we primarily consider the cytoarchitectural cortical regions of the amygdala as limbic cortical output regions. These cortical regions are reciprocally connected with many other archicortical and mesocortical areas. Moreover, we consider the basolateral nucleus to represent both superficial (true cortical) and deep (cortical architecture) cortical amygdaloid areas (Benarroch, 2015a). These basolateral regions can be considered to be “receiving” areas of the amygdaloid complex, and the centromedial (ganglionic or nuclear) region can be considered to be the “emitting” areas (Benarroch, 2015a). The stria terminalis connects this nuclear amygdala to the diencephalon, and from there, the anterior dorsomedial frontal areas can be reached, although the majority of output from the limbic basal ganglia flows to the brainstem. In spite of this, the amygdala also affects the motor output by inducing the drive to seek food, warmth, comfort, etc., or to escape from pain, thirst, distress, etc. (Sewards and Sewards, 2003).

Two types of cortical-subcortical circuits may therefore be distinguished: extrapyramidal and limbic. These systems have different first step relay stations: the extrapyramidal circuit includes the caudate nucleus, putamen, and nucleus accumbens core (NAcbC), whilst the limbic circuit includes the nuclear amygdala, extended amygdala, bed nucleus of the stria terminalis (these three together are the original “extended amygdala” according to Cassell et al., 1999; Heimer and Van Hoesen, 2006), and the nucleus accumbens shell (NAcbS). The extrapyramidal circuit regulates “rational,” cognitively constructed, skilled behavior, which is often goal-oriented and includes decision making. The limbic circuit regulates emotional (instinctive and automatic) behaviors, which are often defensive, and this regulation includes (attentive) salience. The two systems can inhibit or activate, depending on the situation. It is generally accepted that the prefrontal cortex (PFC) selects the appropriate response (Stuss and Knight, 2002; Fuster, 2008). The dorsolateral PFC is particularly important for controlling rational responses, and the medial PFC for controlling emotional responses. Within the medial PFC, the orbitofrontal cortex (OFC) plays a particularly noteworthy role because it is essential for regulating the direction of motivation (Zald and Rauch, 2006).

Behavior can be a reaction to an influence in the environment, and can also be generated by the individual: to enable this reactive vs. proactive behavior, motivation comes into play (Rolls, 1999; Zald and Rauch, 2006). Three stages of behavioral motivation can be distinguished: general motivation, taking initiative, and selective precedence-conveying (via inhibition). The OFC plays a significant role in regulating these processes by delivering input to the ventral striatum, the anterior cingulate cortex, and the amygdala. Although the extrapyramidal and limbic circuits regulate two different types of behavior (cognitive and intuitive, respectively), the individual must generally be highly motivated in order to express these conducts. This motivation requires the involvement of two specific structures: the NAcbC and the NAcbS (Groenewegen and Trimble, 2007; Dalley et al., 2008; Loonen and Stahl, 2011). The NAcbC motivates the individual toward behavior that may lead to a feeling of reward, while the NAcbS motivates the individual toward behavior that may lead to escape from feeling misery (Loonen and Ivanova, 2016a). When strong stimulation of these motivations suddenly ceases, the individual experiences feelings of pleasure (NAcbC) or feelings of happiness (NAcbS), therefore we can distinguish between circuits that regulate pleasure and those that regulate happiness. These circuits overlap, but essentially differ from the earlier mentioned limbic and extrapyramidal circuits. Moreover, they reciprocally influence each other (in a yin-and-yang-like fashion). In clinical depression, both circuits seem to be dysfunctional. Low activity in the reward-seeking system results in the inability to experience pleasure (anhedonia), and in a lack of energy as well as indecisiveness. High levels of activity in the misery-fleeing system results in continuous worrying, negative expectations, and dysphoric feelings. “Misery” refers here to experiencing all types of aversive circumstances like danger, heat, cold, loss or pain, hence not only “misery” in an anthropomorphic sense.

The activities of the NAcbC and NAcbS are controlled by monoaminergic nuclei within the midbrain, which represents the third level of regulation. These nuclei transmit signals through dopaminergic (ventral tegmental area, VTA), adrenergic (norepinephrine, locus coeruleus), and serotonergic (5-hydroxytryptamine, raphe nuclei) tracts. In addition to their direct regulation of the NAcbC and/or NAcbS (Loonen and Ivanova, 2016a; Loonen et al., 2016), the monoaminergic nuclei regulate the activity of other, first relay-station basal ganglia and important parts of other areas in the forebrain. Therefore, behavioral output is controlled at three levels within the brain. The highest level is the cerebral cortex (isocortex, limbic cortex, superficial and deep corticoid amygdala, and hippocampal complex); the second level is the subcortical forebrain (dorsal striatum, ventral striatum, and extended amygdala), and the third level is the midbrain (monoaminergic regulation centers).

A fourth level of regulation would be the coupling between cerebral cortex and midbrain. An important pathway in this respect is including a very ancient structure within the diencephalon: the habenula (from the Latin, little rein), which is located in the dorsomedial portion of the thalamus (Benarroch, 2015b). The habenular nuclei are paired structures and belong to the epithalamus, which also harbors the pineal gland and the stria medullaris. It consists of a larger lateral (LHb) and a smaller medial (MHb) division, each of which consist of a complex set of subdivisions (Klemm, 2004; Benarroch, 2015b). The habenula is a prominent component of the brain of lamprey, which is believed to have a forebrain comparable with our earliest vertebrate ancestors living about 560 million years ago (Loonen and Ivanova, 2015). In lamprey, the activity of the LHb is regulated by habenula-projecting globus pallidus (GPh). We have hypothesized that the same may be true in humans. Within the extrapyramidal circuits, the human homolog of GPh may be localized within the border region of the globus pallidus (GPb) (Stephenson-Jones et al., 2013) and the ventral pallidum (VP) (Hong and Hikosaka, 2013), although the limbic circuits may also contain an equivalent of the GPh. In this paper, we will present evidence supporting the hypothesis that the bed nucleus of the stria terminalis (and possibly neighboring septal areas) might be the limbic human homolog of GPh using the work of Moreno et al. (2012) as a start point. We will also describe the evolution of the amygdaloid complex and hippocampus in relation to the modern human cerebral cortex. This will lead to an improved model showing how reward-seeking and misery-fleeing behavior is regulated in humans, and how dysfunction of these regulatory circuits may cause mental disorders.

## Evolution to the human forebrain

**Figure 2:**
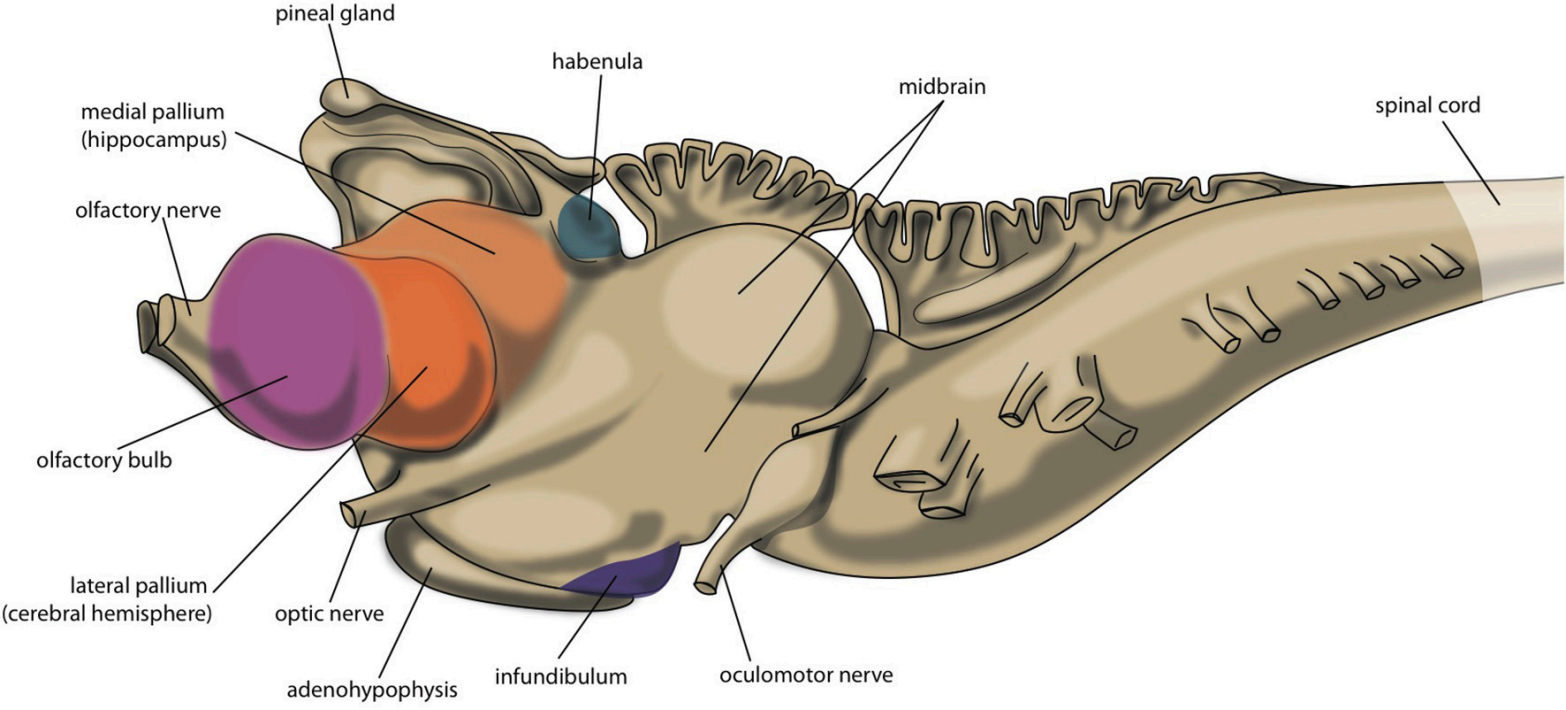
**Central nervous system of lamprey**.

An important reason for us to become interested in the embryology, connectivity and neuroanatomy of primitive vertebrates is the scientific notion that their primitive brains may reflect earlier evolutionary stages of the current human brain. Hence, the brains of Agnatha (jawless fishes represented by hagfishes and lampreys), Chondrichthyes (cartilaginous fishes: eg. sharks and rays, or ratfishes and chimeras), Osteichthyes (bony fishes divided into ray-finned fishes and the lobe-limbed vertebrates), Amphibia (amphibians represented by toads and frogs), Reptilia (reptiles: turtles, lizards, snakes) and Aves (birds) and Mammalia (mammals: opossums, mice, rats, cats) including Primates (primates: monkeys, apes, humans) correspond to the brains of our human ancestors from about 560 million years ago until the present (Moreno and González, 2011). A special position is taken by lungfish (a lobe-finned fish, closest ancestor of all tetrapods) and turtles as being comparable to ancient creatures which appear to be the most precisely positioned within this evolutionary line, while more recent reptiles and birds (sauropsids) appear to derive from another line of turtle-like ancestors rather than mammals (Butler et al., 2011; Moreno and González, 2011; Montiel et al., 2016). The very first vertebrate is considered to be an animal comparable with modern lamprey; this animal has a head containing a brain and has vertebrates, but not yet a lower jaw. The lamprey's central nervous system consists of spinal cord, brainstem, forebrain and olfactory bulbs, but lacks a well-developed cerebellum (Nieuwenhuys and Nicholson, 1998; Loonen and Ivanova, 2015). Its forebrain consists of olfactory bulbs, medial and lateral pallium, subpallium and diencephalon (extensive thalamus). The lateral pallium forms a primitive hemisphere (Figure 2), although some controversy exists over how to divide it into different fields. Wicht and Northcutt (1998) have studied the connections of the hagfish pallium, an ancestor of lamprey, and give several reasons for why this pallium is only a homolog of one or two cortical areas of higher developed craniates. In line with this, Medina and Abellán (2009) state that the dorsal pallium does not appear to be present in the lamprey, and was likely to have been absent in the first vertebrates. The dorsal pallium gives rise to the majority of the mammalian cerebral neocortex (Medina and Abellán, 2009). Ocaña et al. (2015) have recently obtained evidence for functional connectivity between the dorsal and dorsomedial part of lateral pallium and certain brainstem motor centers (pretectum, tectum, midbrain tegmentum and locomotor region, and reticulospinal cells) in lampreys. The brainstem structures they described may correspond to brainstem structures for smooth pursuit eye movements (Mustari et al., 2009) and the pedunculopontine nucleus (PPN, Benarroch, 2013; Gut and Winn, 2016). Both structures are connected with the cerebral neocortex in primates (Matsumura et al., 2000; Aravamuthan et al., 2007, 2009; Mustari et al., 2009). The most likely explanation for the findings of Ocaña et al. (2015) would be that the part of the lateral pallium (dorsolateral pallium) which hosts the cell bodies of pallio-pretecto-midbrain neurons develops into the frontal motor cortex in primates. Hence, the earliest vertebrate human ancestor (comparable with modern lamprey) must already have had a dorsal pallium which in mammals gives rise to the cerebral neocortex. The medial pallium is considered to evolve into the hippocampal complex in all tetrapods, and perhaps all jawed vertebrates (Medina and Abellán, 2009). The ventral pallium which appears to be present in all vertebrates, but also in hagfish, is related to olfactory structures and included as part of the amygdala in tetrapods (Medina and Abellán, 2009).

## Evolution of the ancient pallium to the human cerebral cortex

Studying the development of the human cerebral cortex during subsequent steps of evolution brings a few typical problems. Firstly, in ray-finned fishes, the hemisphere is everted instead of evaginated, which results in an entirely different topography (Moreno and Gonzaléz, 2007; Nieuwenhuys, 2009). Secondly, in sauropsids, the neocortex developed in another direction than in mammals: sauropsids have a large dorsal ventrical ridge, a nuclear structure with no clear counterpart in other vertebrates (Medina and Abellán, 2009). Finally, a large part of the pallium in mammals is laminated, including the hippocampal formation, the neocortex, the olfactory cortex, the entorhinal cortex and the cortical regions of the pallial amygdala (Medina and Abellán, 2009). Other pallidal structures (claustrum, basolateral amygdala) remain unlaminated. Although some areas in certain sauropsids possess a three-layered structure, the pallium in non-mammals is primary organized in a non-laminar fashion. By integrating comparative neuroanatomy with comparative embryology and developmental genetics, much insight has been gained (Medina and Abellán, 2009; Butler et al., 2011; Montiel et al., 2016).

In human embryos, the future insula is the first cortical structure to develop (O'Rahilly and Müller, 2006; Kalani et al., 2009). The primordial insula is initially located on the free lateral surface of the cerebral hemisphere and adjoins the cortical amygdaloid regions on one side and olfactory cortical regions on the other (O'Rahilly and Müller, 2006; Nieuwenhuys, 2012). This is highly comparable to the position of the dorsal pallium in lamprey hemispheres. According to the Von Baerian theory, the embryos of later-descendent species resemble the embryos of earlier-descendant species to the point of their divergence. So, it may be suggested that the human insula is the most ancient part of the neocortex. In addition, the lamprey has a small but well developed dorsal thalamus (the part forming the proper so-called “thalamus” in humans) which connects the tectum (e.g., somatosensory and viscerosensory information) and optic tract (visual information) with caudal parts of the pallium (mainly hippocampal primordium and subhippocampal lobe, i.e., medial pallium; Nieuwenhuys and Nicholson, 1998).

In amphibians (e.g. the oriental fire-bellied toad), which represent an ancestor of a far later stage of evolution, the dorsal pallium has significantly expanded and covers almost the entire roof of the hemisphere (Roth et al., 2007). Fibers of neurons within these dorsal pallial fields run ipsilateral to other pallial regions (medial, lateral, ventral), septum, and striatopallidum (Roth et al., 2007). However, the projections of the thalamus are certainly not restricted to these new dorsal pallial fields (Roth et al., 2003; Laberge and Roth, 2007; Laberge et al., 2008). Anterior parts of the anuran thalamus project to the medial and dorsal pallium, dorsal and ventral striatum, nuclear amygdala, lateral septum and diagonal band of Broca, while visual information is projected to the hypothalamus and brainstem (Roth et al., 2003). From electrical recording and anatomical labeling experiments, it can be concluded that the anuran dorsal pallium has not yet achieved the equivalent of a human input processing and output generating role (Roth et al., 2003; Laberge and Roth, 2007; Laberge et al., 2008), but is still part of a more extensive “limbic” behavioral control system including almost all pallial and subpallial regions.

In more recent jawed vertebrates, the input to the dorsal thalamus largely increases, and this in turn leads to a significant expansion of the dorsal pallium (Butler, 1994a,b). However, this expansion occurs along different lines in non-synapsid (reptiles, birds, turtles) and synapsid (mammals) animals (Butler, 1994b; Montiel et al., 2016). The dorsal thalamus consists of two divisions called lemnothalamus and collothalamus. In non-synapsid animals only the collothalamus with the corresponding lateral dorsal pallial field is largely expanded, while in the synapsid line leading to mammals both the collothalamus with lateral dorsal pallial fields and the lemnothalamus with the corresponding medial dorsal pallial fields developed (Butler, 1994b). The medial part of the lemnothalamic division forms the subicular, cingulate, prefrontal, sensorimotor, and related cortices in mammals. The lateral part forms the striate (visual) cortex. Specific fields within the collothalamic lateral division of the dorsal pallium form the extrastriate visual, auditory, secondary somatosensory, and related cortices in mammals (Butler, 1994b). The expansion just described probably resulted in a total displacement of ventral and medial pallial fields. The medial pallium became the hippocampus and the ventral pallium became the most caudal edge of the frontal lobe (including olfactory tubercle) and cortical regions of the amygdaloid complex in the temporal lobe. The reason why the pallium of this ancestor with a turtle-like brain developed differently in synapsid and non-synapsid evolutionary lines is an interesting subject for future research. In our opinion, the largest advantages of the structure of the human neocortex in controlling behavior became evident too late during evolution to be a selection advantage in this turtle-like stage. Perhaps, it is related to the development of relevant senses.

Secondary to the synapsid development of the dorsal pallium, as described previously, the lamination of the cerebral cortex occurred (Medina and Abellán, 2009; Rakic, 2009). This lamination is absent in non-mammals, and is not restricted to new, originally dorsal, pallial fields (Medina and Abellán, 2009). Due to this lamination, the human neocortex consists of horizontal layers intersected by vertical (or radial) columns which are stereotypically interconnected in the vertical dimension and comprise of processing units (Rakic, 2009; Loonen, 2013). This can be considered to allow more adequate processing than in a non-laminated organization. The expansion and elaboration of the cerebral neocortex during evolutionary development from primitive mammals to humans resulted in the formation of new areas with new connections creating numerous extensive networks regulating different, new or more sophisticated types of behavior (Rakic, 2009). However, the majority of this progress is of a relatively recent evolutionary date.

## Evolution of subcortical structures

**Figure 3:**
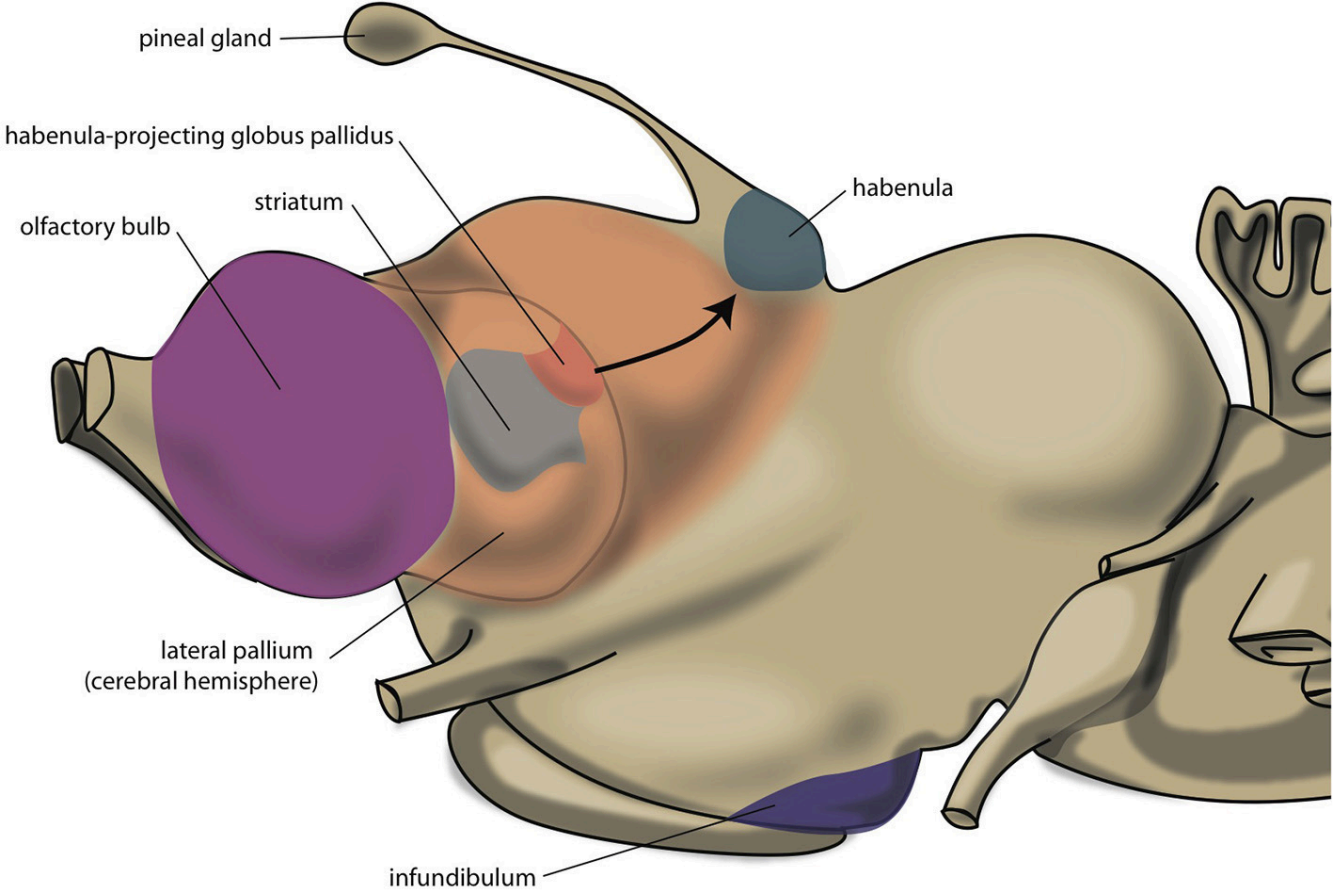
**Position of striatum and habenula-projecting globus pallidus of lamprey**.

**Figure 4:**
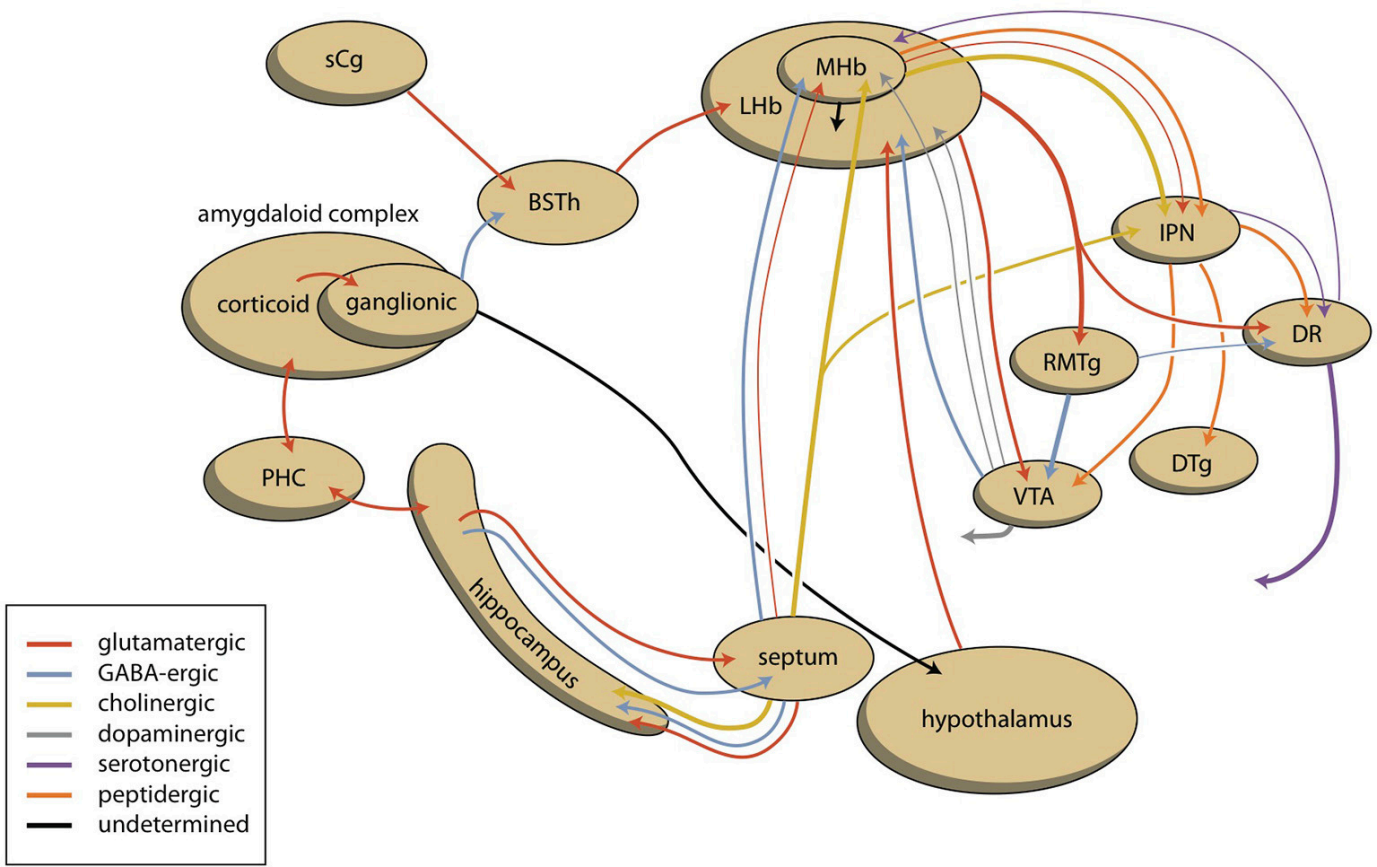
**Scheme showing the connectivity of limbic (cortical) system to the midbrain through the habenular complex**. BSTh, habenula-projecting part of bed nucleus of the stria terminalis; DR, dorsal raphe nucleus; DTg, dorsal tegmental nucleus; IPN, interpeduncular nucleus; LHb, lateral habenula; MHb, medial habenula; PHC, parahippocampal cortex; RMTg, rostromedial tegmental nucleus; sCg, subgenual cingulate gyrus; VTA, ventral tegmental area (for details see Supplementary Material).

The lamprey telencephalon can be divided into a dorsal, pallial region and a ventral, subpallial region. This subpallial part largely consists of striatum, septum and preoptic area (Nieuwenhuys and Nicholson, 1998). Grillner's group has demonstrated that the complete basal ganglia circuitry is already present in these phylogenetically oldest vertebrates (Stephenson-Jones et al., 2011, 2012). Moreover, these animals possess a subpallial structure (Figure 3), the habenula-projecting globus pallidus (GPh), which has an essential role in selecting behaviors that are either rewarding and should be continued, or are not rewarding and should be abandoned (Stephenson-Jones et al., 2013). We have hypothesized that the lampreys' striatal subpallium is included in the nuclear amygdala of tetrapods (Loonen and Ivanova, 2015). Some controversy exists, however, concerning the fate of the GPh in more recent vertebrates. Lamprey also have an epithalamus which is very similar to its homolog in more modern animals (Loonen and Ivanova, 2015) which includes the output structures of the habenula via the fasciculus retroflexus to the midbrain nuclei (Figure 4).

It was formerly believed that the forebrain (especially its basal ganglia) underwent important changes during the evolution from anamniotes (lampreys, fishes, amphibians) to amniotes (reptiles, birds, and mammals). However, the organization of the basal ganglia is more conserved than previously thought (González et al., 2014). The two main components of the basal ganglia develop embryologically from two different areas: the lateral ganglionic eminence (giving rise to striatum) and the medial ganglionic eminence (giving rise to pallidum). During embryological development, different genes are brought to expression in order to regulate the regionalization of these two areas. In particular, the transcription factor Nkx2.1 is expressed in the medial ganglionic eminence (and not the lateral one), and the expression of this gene in combination with that of others has been used to characterize pallidal basal ganglia in embryos of amniotes and anamniotes (González et al., 2014). These authors were able to identify striatal and pallidal regions in cartilaginous fishes, ray-finned fishes, lungfishes and amphibians. However, in the subpallium of lamprey, a pallidal region could not be identified because these animals lack an Nkx2.1-expressing zone: it was suggested that the pallidum is still absent in agnathans, and first appeared during or after the transition from jawless to jawed vertebrates (Moreno et al., 2009). Since Stephenson-Jones et al. (2011, 2012) have demonstrated the existence of a complete extrapyramidal circuitry in the subpallium of lamprey, the proposal of Moreno et al. (2009) has to be rejected. In lamprey, pallidal structures obviously can exist in spite of the absence of expression of Nkx2.1 during the embryonic development of this structure.

The nuclear part of the amygdaloid complex is another derivative of the lateral ganglionic eminence, and the development of the amphibian amygdaloid complex has been studied in detail by Moreno and González (2003, 2004, 2005, 2006). Within the anuran forebrain, the striatum (anterior) is continuous with the central and medial amygdala (posterior), and is clearly separated from dorsal/ventral pallidum, and from the bed nucleus of the stria terminalis and septum (Moreno and González, 2006). In humans, the bed nucleus of the stria terminalis is continuous with the extended amygdala on one side and with the shell part of the nucleus accumbens (NAcbS) on the other (Loonen, 2013; Loonen and Ivanova, 2015). It should be noted that the original concept described the centromedial amygdala and bed nucleus of the stria terminalis both as being part of the extended amygdala (Cassell et al., 1999; Heimer and Van Hoesen, 2006). This is also supported by embryonic migration of neuronal cells generated in the hypothalamus to the nuclear amygdala as well as the bed nucleus of the stria terminalis (García-Moreno et al., 2010). The investigations made clear that the organization of the ancestral tetrapod (amphibian-like) amygdaloid complex is retained within more recent ancestors (Moreno and Gonzaléz, 2007). Evolution of the anamnio-amniotic (mammalian) “extrapyramidal” striatum probably occurred in a modular sense when a more lateral part of the striatum was added each time a neocortical area with a new function was added to the expanding neocortex (Grillner et al., 2013; Robertson et al., 2014). Probably in parallel to this neostriatum, the corresponding “extrapyramidal” globus pallidus and substantia nigra (reticulata and compacta) developed. As described above, the amygdaloid complex derives from pallial and subpallial territories. Pallial (corticoid) structures include the cortical amygdala (olfactory and vomeronasal) and the basolateral complex deep to it (Martínez-García et al., 2002). These pallial components originate from lateral and ventral pallial regions, and are also maintained during evolution of amniotic vertebrates (Martínez-García et al., 2002; Moreno and Gonzaléz, 2007).

An important discovery during the study of the embryological development of anuran basal ganglia was the finding that the bed nucleus of the stria terminalis (BST) and part of the septum are also of pallidal instead of striatal origin (Moreno et al., 2012; González et al., 2014). This is interesting because the BST is a suitable structure for the execution of the limbic component of the lampreys' habenula-projecting globus pallidus. The architecture and connectivity of the rat BST has been studied in detail by Larry Swanson and collaborators (Ju and Swanson, 1989; Ju et al., 1989; Dong et al., 2000, 2001a; Dong and Swanson, 2003, 2004a,b, 2006a,b,c). It becomes evident that the rat BST is an extremely complex set of nuclei which can be separated into dorsal, lateral and ventral areas (Ju and Swanson, 1989). These nuclei receive input from the central amygdaloid nucleus (innervating various parts of the anterior BST division) and medial amygdaloid nucleus (preferentially innervating the posterior BST division), but not from the superficial and deep corticoid nuclei of the amygdala (Dong et al., 2001b). The conclusion is that BST is a rostral differentiation of the pallidum receiving massive GABAergic input from centromedial amygdala and giving GABAergic output to brainstem motor systems and thalamocortical re-entrant loops (Dong et al., 2001b). Dense peptidergic transmission from and to rat BST became evident when studying its' chemoarchitecture (Ju et al., 1989). The organization of the projections from and to the separate BST nuclei is too complex to be described within the context of this overview; but viewed broadly, BST posterior division cell groups share massive bidirectional connections with the medial amygdaloid nucleus and other amygdaloid components of the accessory olfactory system, and send massive projections to hypothalamic control centers regulating reproduction and defense (Dong and Swanson, 2006a). The BST anterolateral group projects to the ventral autonomic control network to the midbrain structures modulating the expression of orofacial and locomotor somatosensory responses, and to the ventral striatopallidal system. This suggests that the anterolateral group is primary involved in appetitive feeding (eating and drinking) behavior (Dong and Swanson, 2006a). In rats the lateral habenula receives very few fibers from these BST areas. However, the anteromedial area (BSTamg), and even more extensively the dorsomedial nucleus (BSTdm) of anteromedial BST division, projects to the medial, resp. caudal regions of the lateral habenula (Dong and Swanson, 2006a,c). Lateral habenula afferents from the BST are also described by Felton et al. (1999), but these authors give few specifics and the neurochemical characteristics of these connections have not been elucidated. In our opinion, it is very possible that the anteromedial division of the rat BST contains glutamatergic neurons which run parallel to the lateral habenula and have a similar function to lamprey GPh neurons in inhibiting the activity of dopaminergic midbrain nuclei, resulting in inhibition of behavior when its positive results are disappointing (comparable to anti-reward sensing). Further, the anterior BST division receives input from the hippocampus (ventral subiculum) and infralimbic cortex (comparable with the human subgenual anterior cingulate cortex, Brodmann Area 25, BA25). It should be borne in mind that the ventral subiculum projects substantially to the infralimbic area (Dong et al., 2001b); this connectivity probably corresponds to the cortical input to the habenula-projecting globus pallidus.

Within the extrapyramidal system, it has been suggested that the GPh has been conserved in non-human primates as the border region of the globus pallidus (GPb) (Stephenson-Jones et al., 2013). These GPb cells probably correspond to lateral habenula (LHb)-projecting excitatory neurons which respond to reward or no-reward indicators (Hong and Hikosaka, 2008). These authors confirmed the results of Parent et al. (2001), who distinguished between two types of projection neurons in the border of the internal pallidum of primates, one acting upon thalamic and brainstem premotor neurons, whereas the other type acted upon LHb neurons. The role of LHb-projecting GPb neurons was confirmed recently in primates, but Hong and Hikosaka (2013) also found that LHb-projecting neurons originated within the ventral pallidum (VP). The ventral pallidum (VP) receives most input from the nucleus accumbens (ventral striatum) (Groenewegen and Trimble, 2007) and projects to the LHb (Haber et al., 1993). However, the majority of descending efferent projection from the ventral pallidum in monkeys terminates primarily in the subthalamic nucleus and the adjacent lateral hypothalamus, and in the substantia nigra. Ventral striatum and ventral pallidum are part of the ventral cortical-striato-pallido-thalamo-cortical circuit regulating motivation to reward-seeking (Nucleus Accumbens Core; NAcbC) and misery-fleeing behavior (Nucleus Accumbens Shell; NAcbS). Dorsal striatum and global pallidus are primary involved in decision-making and the control of voluntary motions (Grillner and Robertson, 2015). The cortical input to the NAcbS primarily originates within the medial prefrontal cortex and the medial edge of the OFC, and cortical projections to the NAcbC within the rest of the OFC (Haber et al., 1995). This was specified by Ferry et al. (2000) who describe that the most ventromedial parts of the caudate–putamen and the core of the nucleus accumbens receive projections from areas of the so-called “medial prefrontal network,” which include the medial prefrontal cortex and some of the more caudal orbital areas, whereas the central parts of the caudate nucleus and putamen receive projections from areas of the “orbital prefrontal network” which include the remaining, mostly more rostral located, orbitofrontal areas. Hence, in non-human primates the mediocaudal OFC most heavily projects to the NAcb, and the rostrolateral OFC to central parts of the caudate and putamen. In particular, the central parts of the caudate and putamen are connected to the LHb (Hong and Hikosaka, 2013). Therefore, the rostrolateral OFC may correspond to at least one of the cortical areas giving input to the GPb; the cortical areas giving input to the habenula-projecting part of the ventral pallidum (VPh) remain to be determined.

**Figure 5:**
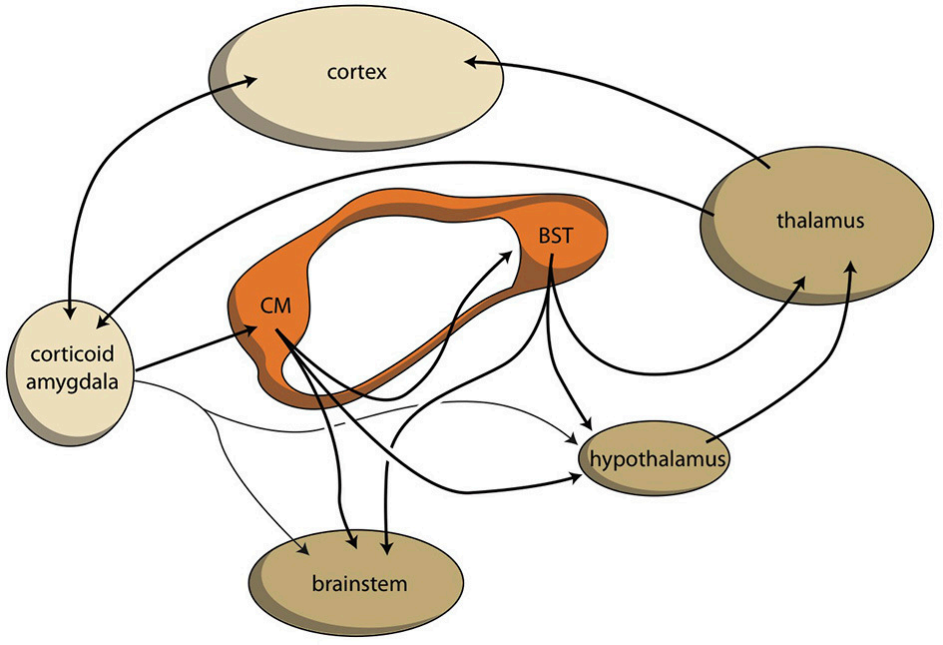
**Limbic cortical-subcortical regulatory circuit**. BST, bed nucleus of the stria terminalis; CM, centromedial amygdala; orange, extended amygdala; dark yellow, diencephalon, and brainstem; light yellow, corticoid amygdala, and neocortex.

Similar to the organization of extrapyramidal cortical-subcortical circuits, within the amygdala a cortical-subcortical circuit also exists, although the latter is constructed in a more complex manner due to its ancienity: the system developed long before the neocortex existed. To simplify, we propose that the corticoid regions of the amygdala should represent the primary limbic cortex. These corticoid regions are bi-directionally connected, with many neocortical areas. The superficial (cortical) and deep (basolateral) corticoid regions of the amygdaloid complex can be considered to be the cortex, and the centromedial (nuclear) region can be considered as being the striatum of the amygdaloid complex. In the earliest vertebrate ancestors, the striatopallidum directly drives autonomic and motor control centers in the lower diencephalon and brainstem (Loonen and Ivanova, 2015). In lamprey, very limited connectivity exists between pallial (cortex) areas and diencephalic and brainstem control centers. This is also true within the corresponding system in mammals: only light connectivity has been found between corticoid amygdalar areas and the hypothalamus or brainstem (Pitkänen, 2000). The stria terminalis connects the “striatal” centromedial amygdala with its corresponding “pallidum” (i.e., bed nucleus of the stria terminalis), and also directly with the hypothalamus and brainstem (Pitkänen, 2000). Although the majority of output from the limbic basal ganglia flows to the brainstem, connectivity also exists via the (dorsal) thalamus with the cerebral cortex. This is true for the output of the bed nucleus of the stria terminalis (Dong et al., 2001b; Swanson, 2003), and for the output of the hypothalamus, which is probably related in order to affect the motor output of higher vertebrates, including humans, by inducing the drive to seek food, warmth, comfort, etcetera, or to escape from pain, thirst, misery, etcetera (Sewards and Sewards, 2003). This results finally in a limbic cortical-subcortical circuit that is more complex, but nevertheless essentially similar, to the well-known extrapyramidal system, provided that one realizes that the cerebral neocortex was included within the circuit on a later evolutionary moment (Figure 5).

## Evolution of the amygdaloid complex

The endbrain (telencephalon) of the very first vertebrates can be considered to be the evolutionary starting point of human amygdaloid complex. Its pallium largely consisted of ventral, lateral and medial fields; its dorsal pallium was not contributing to a very significant extent. The subpallium contained a striatopallidal complex for motor control, and habenula-projecting globus pallidus for decision making. During the evolutionary period in the development to becoming an amphibian-like ancestor, the dorsal pallium developed to a significant extent, but it can still be considered an extension of the medial pallium. This can be concluded from its connectivity with other pallial and subpallial structures, as well as from its input received from the dorsal thalamus. The medial pallium later developed into the hippocampus. At a subpallidal level, the primitive striato-pallidal complex becomes, respectively, the nuclear amygdala and the bed nucleus of the stria terminalis. This limbic striatopallidal structure will later become the human extended amygdala (as defined by Cassell et al., 1999; Heimer and Van Hoesen, 2006). Next to the amygdaloid complex, a new anterior striatopallidal complex arises in amphibians which will form the extrapyramidal system in our mammalian ancestors. In our opinion it actually took until the evolution of our mammalian ancestors before the dorsal pallium was essentially actually transformed into the current neocortex. The massive growth of this neocortex resulted in a C-shaped and outside-inward curving of the cerebral hemispheres. The medial pallium became the hippocampus, and the ventral pallium the superficial and deep corticoid amygdala. This means that almost the entire cerebral hemisphere is of quite recent origin; this is probably also true for the limbic cortical-subcortical-neocortical connectivity we have previously suggested (Loonen and Ivanova, 2015, 2016a). Corticoid amygdaloid output reaches the hypothalamus and brainstem (to a minor extent directly and) largely along nuclear amygdala (striatal amygdala) and the bed nucleus of the stria terminalis (pallidal amygdala). This results directly from the regulation of vegetative and motor behavior by striatum instead of by pallium in lamprey (Loonen and Ivanova, 2015). The human frontal neocortex, however, is reached through connectivity with the dorsal thalamus; this last connectivity must have developed later during the evolutionary development of the mammalian forebrain. The amygdaloid equivalent of the habenula-projecting globus pallidus is probably localized within the bed nucleus of the stria terminalis. This arrangement would be beneficial for survival because the entire regulatory system would be maintained within the amygdalo-hippocampal complex and the capacity of the thalamocortical system would be added to this without replacing anything of the acquired capabilities. This allows the cerebral cortex to develop into a very sophisticated regulatory structure without endangering the vital functions controlled by the more primitive amygdalo-hippocampal system.

## Connectivity of the habenula

**Figure 6:**
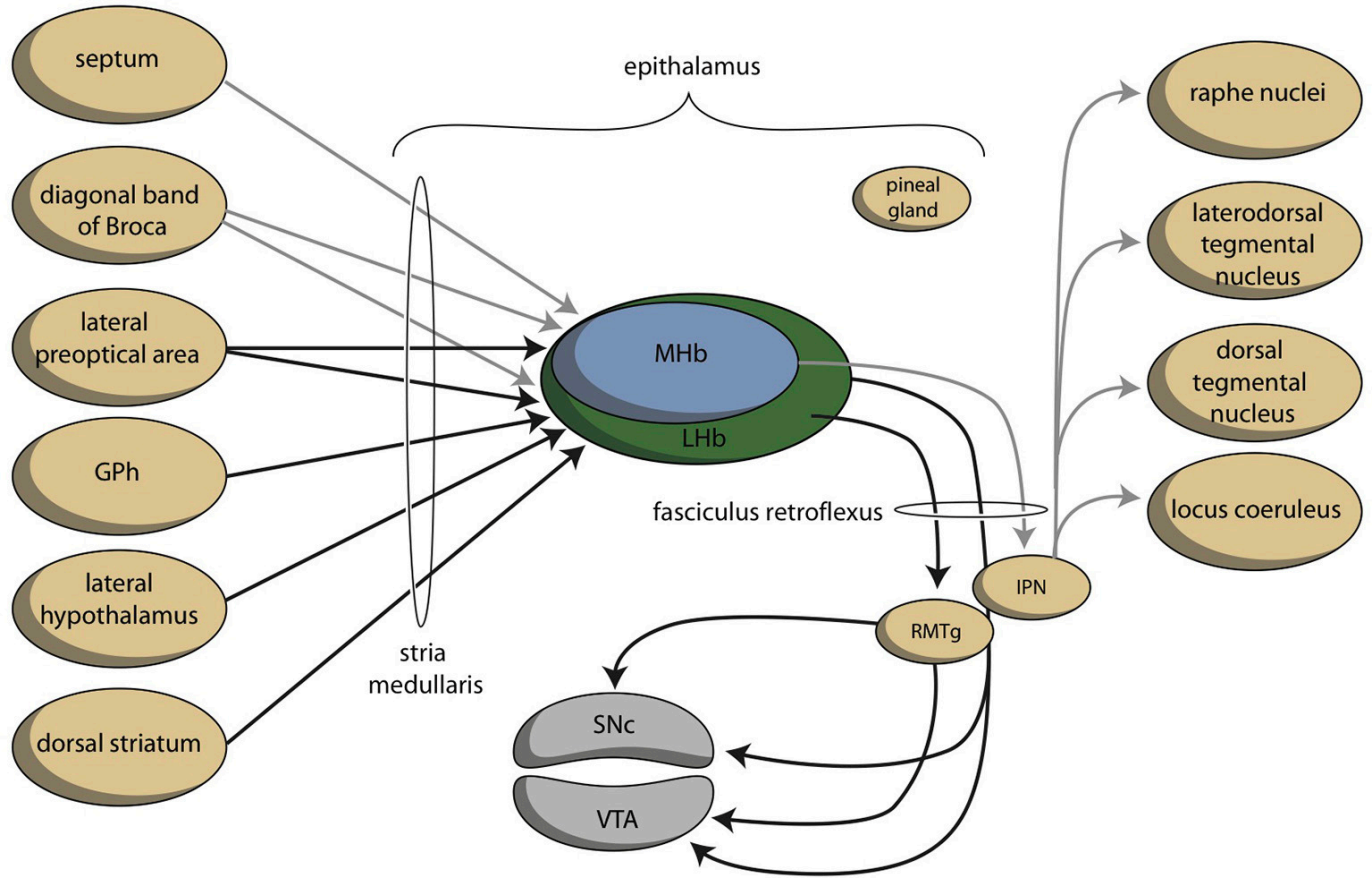
**Simplified representation of the connectivity through the epithalamus (adapted from Hikosaka, 2010)**. GPh, habenula-projecting globus pallidus; IPN, interpeduncular nucleus; RMTg, rostromedial tegmental nucleus; SNc, substantia nigra, pars compacta; VTA, ventral tegmental area. GPh depends upon the cortical-subcortical circuit being considered. GPh, localized within the bed nucleus of the stria terminalis in the limbic circuit, within the ventral pallidum concerning the motivational circuit and within the globus pallidus (border region, GPb) within the extrapyramidal circuit.

The stria medullaris is the main habenular input and the fasciculus retroflexus the primary output structure of the habenula (Figures 4, 6) (Sutherland, 1982; Klemm, 2004; Bianco and Wilson, 2009; Benarroch, 2015b). The septum, particularly the medial septum and the adjacent nucleus of the diagonal band of Broca, is the major source of afferents of the MHb (Klemm, 2004; Benarroch, 2015b). This input is largely cholinergic and GABAergic (Benarroch, 2015b). Moreover, the MHb receives dopaminergic input from the ventral tegmental area and adrenergic (norepinephrine) input from the locus coeruleus (Bianco and Wilson, 2009; Benarroch, 2015b). The LHb receives glutamatergic afferents primarily from the preoptic area, lateral hypothalamus, the entopeduncular nucleus (rodent analog of globus pallidus in primates), and from anterior cingulate and the medial prefrontal cortex (Benarroch, 2015b). Moreover, the LHB also receives strong GABAergic innervations (Poller et al., 2013) from various brain regions, including, for example, the entopeduncular nucleus, the ventral tegmental area, and the nucleus accumbens. The LHb additionally receives dopaminergic innervation from the ventral tegmental area, serotonergic innervation from the medial raphe nucleus, and adrenergic input from the locus coeruleus, next to an unique population of inhibitory ventral tegmental area neurons that synthesize both dopamine and GABA (Stamatakis and Stuber, 2012; Stamatakis et al., 2013; Benarroch, 2015b). Although the MHb is connected to the LHb, there is no connection from the LHb to the Mhb (Kim and Chang, 2005).

Information processed by the LHb and the MHb is transmitted through the fasciculus retroflexus axon bundle to midbrain monoaminergic nuclei, such as the dopaminergic ventral tegmental area and substantia nigra pars compacta, and the serotonergic raphe nuclei (Hikosaka, 2010). The fasciculus retroflexus is divided into two regions: the outer region originates in the LHb and projects mainly to the rostromedial tegmental nucleus, next to numerous monoaminergic nuclei in the mid and hindbrain (Bianco and Wilson, 2009). The rostromedial tegmental nucleus, which is also named as the “tail” of the ventral tegmental area, is a small nucleus that contains mainly inhibitory GABAergic cells and thereby regulates activity of the ventral tegmental area/substantia nigra pars compacta and the dorsal raphe nucleus (Benarroch, 2015b). LHb neurons also directly target the dopaminergic ventral tegmental area (Lammel et al., 2012) and substantia nigra pars compacta themselves, as well as the serotonergic median and dorsal raphe nucleus, cholinergic laterodorsal tegmentum, and noradrenergic locus coeruleus (Herkenham and Nauta, 1979).

The inner area of the fasciculus retroflexus originates in the MHb, and projects to the interpeduncular nucleus (Sutherland, 1982; Klemm, 2004; Bianco and Wilson, 2009; Benarroch, 2015b). The MHb contains both cholinergic neurons (in its ventral two-thirds) and dorsally located substance P-containing neurons, which innervate the ventral and dorsal vs. the lateral interpeduncular nucleus respectively (Artymyshyn and Murray, 1985; Contestabile et al., 1987). This neuronal pathway is highly conserved across various species (Broms et al., 2015). The MHb is also the main source of the input of the interpeduncular nucleus (Morley, 1986; Klemm, 2004; Bianco and Wilson, 2009), although cholinergic fibers may also originate in the posterior septum (Contestabile and Fonnum, 1983; Fonnum and Contestabile, 1984). The interpeduncular nucleus is a singular, unpaired structure located at the ventral midline of the midbrain (Morley, 1986; Klemm, 2004). The major efferent pathways originating in the interpeduncular nucleus project to the dorsal tegmental nucleus (Morley, 1986), the ventral tegmental area (Klemm, 2004) and the raphe nuclei (Klemm, 2004; Bianco and Wilson, 2009). The interpeduncular nucleus is well known for its widespread projections, both ascending and descending (Klemm, 2004; Morley, 1986). Apart from a low number of serotonergic neurons (continuous with the B8 cell group of the median raphe nucleus) numerous peptidergic neurons (substance P, met-enkephalin, somatostatin) have been identified within the interpeduncular nucleus (Morley, 1986).

## Connectivity of the amygdaloid complex

**Figure 7:**
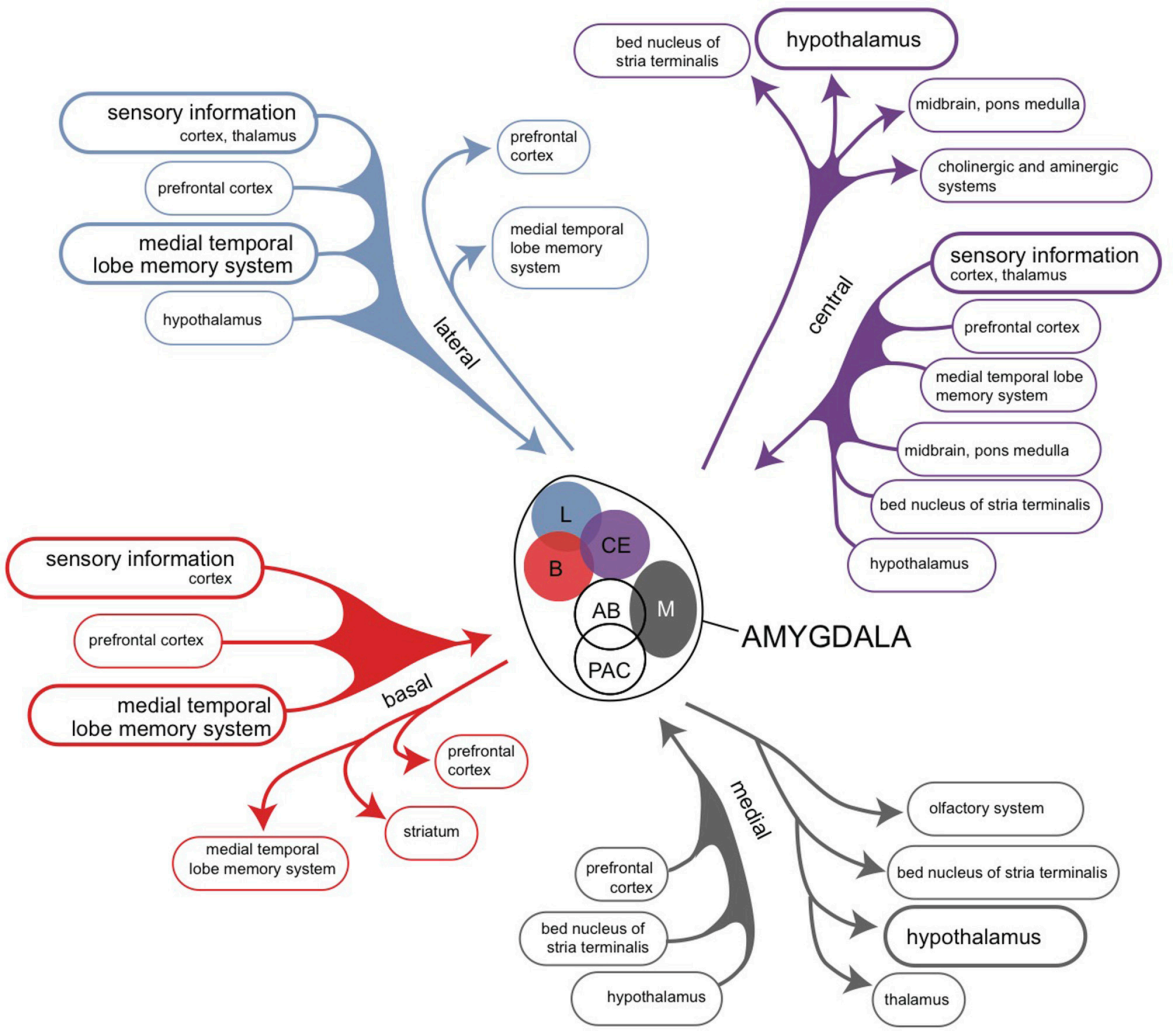
**Overview of the connectivity of the rat amygdaloid complex (Pitkänen, 2000)**.

The amygdaloid complex is a heterogeneous group of 13 nuclei and cortical areas located in the medial temporal lobe just rostral to the hippocampal formation (Freese and Amaral, 2009). The complex can be neuroanatomically divided into “deep nuclei,” “superficial nuclei,” and “remaining nuclei” (Freese and Amaral, 2009). Both the cortical amygdalar nuclei and the basolateral amygdalar nuclear complex, which is located deeper within the amygdaloid complex, have cortex-like cell types (McDonald and Mott, 2016). In contrast, the so called “extended amygdalar nuclei” contain predominantly GABAergic spiny projection neurons, like the striatum (McDonald and Mott, 2016). Each nucleus of the amygdala has a characteristic set of interconnections with other amygdalar nuclei and extrinsic subcortical and cortical brain regions (Figure 7; Pitkänen, 2000; Freese and Amaral, 2009). Within the amygdalar nuclear complex the primary flow is from corticoid to nuclear structures (Benarroch, 2015a).

An unambiguous description of the structure and connectivity of the human corticoid amygdala is hampered by the existence of a predominance of contradictory, confusing and unsubstantiated viewpoints in both in recent as well as older scientific literature (Swanson and Petrovich, 1998; Price, 2003; Swanson, 2003). Further, the connections of the amygdaloid complex have been studied in mammalian animal species (mainly rats, cats, and monkeys) which differ with respect to the extensiveness of their neocortex (Price, 2003). Comparison of these species shows that the large increase in the size of the higher mammalian neocortex causes dominance of projections from and to neocortical areas. Looking again into the connectivity of putative homologs of the amygdaloid complex in our early vertebrate ancestors may be of help: in amphibians, the amygdaloid complex is divided into three components: the vomeronasal amygdala, the olfactory/multimodal amygdala and the autonomic amygdala (Moreno and González, 2006). It should be realized that anuran species probably do not possess a true homolog of the human neocortex (see above). However, the anterior, lateral and medial areas of the anuran amygdaloid complex reveal a connectivity which is roughly running ahead of the connectivity of the neocortex associated amygdaloid complex (Roth et al., 2004; Moreno and González, 2006). This includes output of the later deep nuclear complex and medial amygdaloid nucleus to the medial pallium, which is the eventual hippocampus.

Based on these starting points, three or four components of amygdaloid connectivity can be distinguished (Swanson and Petrovich, 1998; Price, 2003): the accessory olfactory division, the main olfactory division, the autonomic division and the frontotemporal division. As possession of a true human vomeronasal organ, which is originally the main source of input to the accessory olfactory division, is still controversial, the first two may be added together. The frontotemporal division is often primarily associated with strong bidirectional interactions with the prefrontal cortex and hippocampal formation (Pitkänen, 2000; Benarroch, 2015a), but the amygdalohippocampal system can also be considered to be an output channel of the amygdaloid complex. The connectivity of the deep corticoid amygdaloid complex from and to the hippocampal complex is mediated through parahippocampal regions (pre/parasubiculum, entorhinal, perirhinal, and postrhinal/parahippocampal cortices; Witter, 2002). The last three regions are heavily targeted by the olfactory endopiriform cortex, claustrum, basolateral amygdala, medial septum, and dorsal midline thalamic nuclei (Tomás Pereira et al., 2016). Output is primarily provided to the olfactory piriform transition area, basolateral amygdala, claustrum, and dorsal medial thalamic nuclei (Agster et al., 2016). Via the fornix, the hippocampus sends a GABAergic connection to the medial septum and a glutamatergic connection to the lateral septum (Nieuwenhuys et al., 2008; Khakpai et al., 2013). Reciprocally, cholinergic, and to a far less extent GABAergic and glutamatergic, fibers coming from the medial septum-diagonal band of Broca complex run through the fornix to the hippocampus (Khakpai et al., 2013; Nieuwenhuys et al., 2008). Hence, a bidirectional connectivity exists between parahippocampal gyrus and medial septal area with and without including the hippocampus. The medial septum is a primary source of input to the medial habenula.

The four divisions of the amygdaloid system have a more conjoined than separated functional significance. All four divisions regulate in combination with each other in several components of instinctive, emotional behavior. The accessory olfactory component is perhaps somewhat more involved in social behavior related to reproduction, and the autonomic part somewhat more with the regulation of visceral aspects of the emotional response. However, the abundancy of the interactions between separate amygdaloid areas and the extensive mixed connectivity with other brain structures (Pitkänen, 2000; Freese and Amaral, 2009; McDonald and Mott, 2016), mean that separate pathways cannot be clearly distinguished. An important part of this amygdaloid output is delivered, either directly or via hippocampus indirectly, to hypothalamic structures regulating reward-gaining and misery-fleeing behavior (Petrovich et al., 2001; Sewards and Sewards, 2003; Loonen and Ivanova, 2016a). These hypothalamic areas are also reached via the non-centromedial parts of the extended amygdala (including the bed nucleus of the stria terminalis; Dong et al., 2001b; Dong and Swanson, 2003, 2004a,b, 2006a,b,c; Swanson, 2003; Waraczynski, 2016). A major role is played by the deep corticoid complex (mainly basolateral nuclei) in bidirectional interaction with the prefrontal cortical areas, the hippocampal complex as well as sensory cortical areas (Janak and Tye, 2015; Rutishauser et al., 2015; Wassum and Izquierdo, 2015; Benarroch, 2015a; McDonald and Mott, 2016). An essential characteristic of this bidirectional amygdaloid connectivity is the capability to learn from experiences through associative learning, complex response conditioning, episodic memorization, and so on (Benarroch, 2015a). The best description of the function of the amygdaloid complex is to analyze the complex input concerning the actual daily life situation within the individual's biotope (nature, flora, fauna, social circumstances) and to select that sensory input which deserves more attention in order to improve the current chances (misery-fleeing and reward-seeking). The amygdala also receives information about the environment from the sensory thalamus and sensory cortices; the input is compared with memorized information and modulated by programs concerning implicit and explicit behavioral output. This includes the direct inhibition of the amygdala-dependent emotional response when such inhibition expected to be more profitable; this last function is primarily attributed to ventromedial areas of the prefrontal cortex (Kim et al., 2011; Roy et al., 2012; Loonen and Ivanova, 2016a). Traditionally, the amygdala is supposed to induce an emotional response, mainly by giving output to the hypothalamus and brainstem via the centromedial nucleus after this validation process has been completed (Benarroch, 2015a). We want to suggest that a significant part of output is given additionally via the hippocampus and fornix to the medial and lateral septal areas (Nieuwenhuys et al., 2008; Khakpai et al., 2013). After comparison with memorized experiences in the hippocampus and processing within the septal area, the information may reach the medial habenula (MHb). The septum, particularly the medial septum and the adjacent nucleus of the diagonal band of Broca, is the main input to the MHb (Klemm, 2004; Viswanath et al., 2014; Benarroch, 2015b). Although the MHb has been far less extensively studied than the LHb, experimental data support the theory that hyperactivity of the MHb is likely to be associated with depression, anxiety and fear (Viswanath et al., 2014). Hence, the pathway described above, from the corticoid amygdala, via the hippocampus, septal nuclei, medial habenula, and interpeduncular nucleus to ventral tegmental area and raphe nuclei may represent a primary regulation mechanism to increase or decrease the intensity of the emotional response.

In addition, the amygdala affects the activity of the ventral tegmental area through a pathway including the lateral habenula. The striatopallidal (extended) amygdala is heavily (directly and indirectly) connected to the lateral hypothalamus. The activity of the lateral habenula is probably modulated by this pathway. In addition, we want to suggest that anteromedial division of the bed nucleus of the stria terminalis contains the human limbic equivalent of the lamprey habenula-projecting globus pallidus (GPh). This area receives input from GABA-ergic projection neurons originating within the central amygdaloid nucleus (Dong et al., 2001b), and gives output to medial and caudal regions of the lateral habenula (Dong and Swanson, 2006a,c). When this limbic GPh is functioning in a similar manner to lamprey GPh, the amygdala can inhibit reward-seeking behavior by stimulating the pathway, which runs from corticoid amygdala, through central amygdala, anteromedial bed nucleus of the stria terminalis, lateral habenula, and rostromedial tegmental nucleus to ventral tegmental area (Figure 4).

In conclusion, the amygdaloid complex plays an essential role in fear and anger control, perception and attention to relevant sensory input (including, for example, facial expression in order to allow adequate social functioning) by validating this input with respect to their significance for reward-seeking and misery-fleeing behavior. The activity of this emotional response is regulated through a pathway including the habenula, in which two routes can be distinguished: one including hippocampus, septal nuclei and medial habenula and the other including central amygdala, bed nucleus of the stria terminalis and lateral habenula.

## Circuits regulating misery-fleeing and reward-seeking behavior

Previously, we have suggested the existence of two very ancient brain systems regulating reward-seeking and misery fleeing behavior. Reward-seeking behavior leads to the access of necessities like food, water, warmth, comfort, territory, possibilities to reproduce, and so on. Misery-fleeing behavior helps individuals to escape from threats, pain, discomfort and other factors which may decrease chances to stay alive and to have offspring. These two processes are believed to be essential for the continuation both as an individual and as a species. Considering the evolutionary background of these behaviors, the regulation of reward-seeking behavior is more or less obvious. Within lampreys, representatives of our oldest vertebrate ancestors, reward seeking behavior is increased or decreased by a circuit including the lateral habenula. The decision to continue or abandon the reward-seeking behavior is a function of the habenula-projecting globus pallidus. However, the regulation of misery-fleeing behavior is less clear. In this paper we have suggested that the amygdala represents the endbrain of our ancient ancestors. The corticoid amygdala may feed the medial septum and subsequently the medial habenula through the corticoid amygdalar-parahippocampal-hippocampal-fornical-medial septal connection. In addition, the bed nucleus of the stria terminalis contains a habenula-projecting globus pallidus which inhibits reward-seeking behavior once the misery-fleeing behavior is given preference. Both the ventromedial prefrontal and the hippocampal cortex regulate the size of the misery-fleeing response by affecting the corticoid amygdala through bidirectional connectivity.

## Consequences and future research

We have presented a model for the regulation of misery-fleeing and reward-seeking behavior including, respectively, the medial and lateral habenula. This regulatory mechanism is, from an evolutionary perspective, very ancient, and has until now not been very much appreciated considering the influence of neocortical areas on human behavior. In our opinion, this lack of attention is, perhaps, undeserved. The behaviors controlled by the epithalamus are undoubtedly essential for animal life, without the human species being an exception to that rule. In humans, these behaviors are accompanied by vital emotions such as hunger, thirst, fatigue, and sleepiness. A cessation of the urge to display misery-fleeing and reward-seeking behavior may be related to sensing feelings of happiness and pleasure. Alterations in the display of these two types of behaviors are eye-catching components of most, if not all, mental disorders. We have summarized evidence indicating involvement of the mechanisms regulating these two type of behaviors in depression (Loonen and Ivanova, 2016b), bipolar disorder (Loonen et al., submitted), addiction (Loonen et al., 2016), delusions (Loonen and Ivanova, in press), and specific anxiety disorders (in preparation). This is not very surprising considering the primary character of these regulatory processes, and does not conflict with the role of cerebral cortex in inducing the diseased mental state in patients suffering from them. However, the interaction between cortical and subcortical mechanisms may be more important for the regulation of mental processes than is often assumed. Unfortunately, the epithalamus is too small to be studied with common functional imaging techniques. The introduction of more powerful MRI equipment may, however, have changed this situation, although studying the activity of the human homolog of lamprey habenula-projecting globus pallidus with neuroimaging techniques will probably remain too major a challenge. For functional psychopharmacologists, the chemoarchitecture of the input and output of the habenula offers numerous possibilities to specifically affect the motivation of displaying reward-seeking or misery-fleeing behavior.

**Figure 8:**
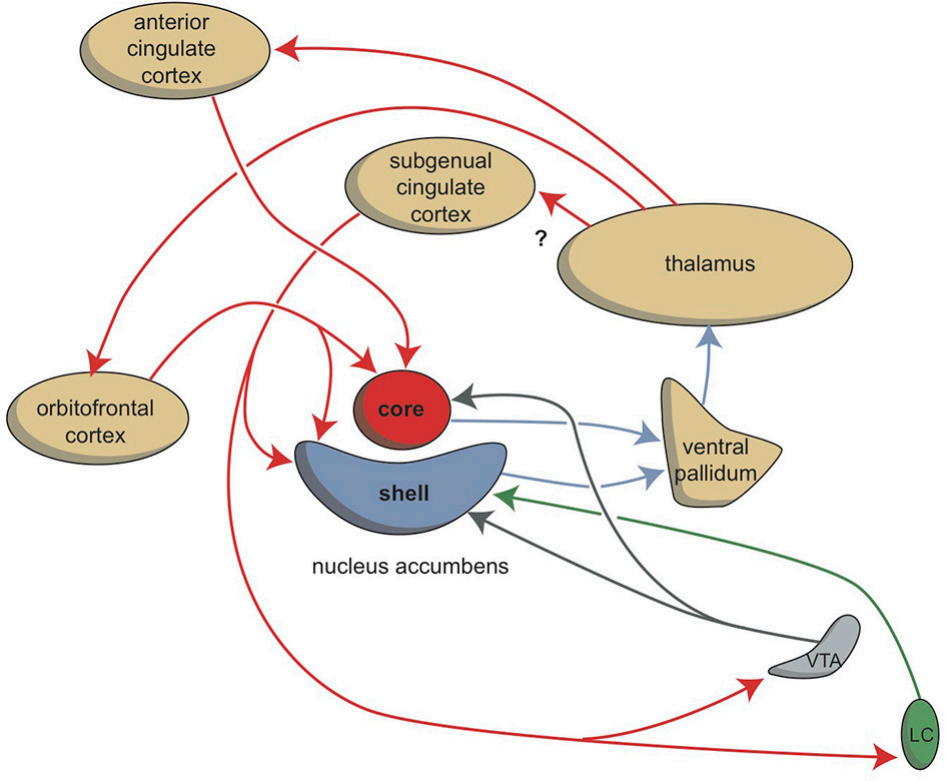
**Cortico-striatal-thalamo-cortical re-entry circuits including core and shell parts of the nucleus accumbens**. The existence of the re-entry circuit including the subgenual cingulate cortex is still hypothetical. LC, locus coeruleus; VTA, ventral tegmental area. Red arrows, glutamatergic; blue arrows, GABAergic; green arrows, adrenergic; gray arrows, dopaminergic.

In this article we have described the organization of the system regulating misery-fleeing behavior. Considering the evolution of the forebrain in vertebrates, we believe that the amygdaloid complex represents the most genuine part of the forebrain initiating and regulating these behaviors. Interactions with parts of the cerebral neocortex must be secondary to this function, since the function of the human neocortex has evolved quite recently within the mammalian developmental process. However, the cerebral cortex with its highly developed recurrent collateral connections between separate cortical areas and its extensive connectivity with the corticoid amygdala, may allow more sophisticated adaptations to environmental challenges by regulating the output of the amygdala than could be achieved by this system alone. Another ancient evolutionary, and therefore primary component, is the interaction of the corticoid amygdala with the hippocampal complex. Next to the memorization of reactions within the corticoid amygdala itself, this may be the primary mechanism by which to relate the necessary reaction to memorized contextual factors, i.e., earlier experiences. However, the hippocampal complex can also considered to be an output pathway connecting the corticoid amygdala via the medial septum with the medial habenula. Activation of this pathway results in a magnification of the misery-fleeing response, and the contribution of the hippocampal complex may be essential to increase or decrease the response depending upon the context. The hippocampus also delivers output to the subgenual cingulate cortex, which may be important for a longer lasting misery-fleeing behavioral state. The main output of the amygdaloid complex is delivered through the extended amygdala (corresponding with the amygdaloid striatopallidum) to the hypothalamus and brainstem. This corresponds to the regulation of motor behavior in our earliest vertebrate ancestors, and is related to both misery-fleeing and reward seeking behavior. This output system was connected with the cerebral neocortex when this structure appeared later during evolution. The bed nucleus of the stria terminalis (amygdaloid pallidum) may also contain a structure comparable with the lateral habenula-projecting globus pallidus of the earliest vertebrates, regulating the balance between reward-seeking and misery-fleeing behavior. It receives input from the central amygdaloid nucleus and the subgenual cingulate cortex. The subgenual cingulate cortex also gives output to the corticoid, and to a lesser extent, the nuclear amygdala (Hamani et al., 2011), but its major role may be related to giving input to the shell part of the nucleus accumbens as part of a still hypothetical limbic cortico-striato-pallido-thalamo-cortical re-entry circuit regulating motivation to exhibit misery-fleeing behavior (Figure 8).

## Author contributions

AL developed the ideas and wrote the manuscript. SI discussed the ideas and commented on the manuscript.

### Conflict of interest statement

The authors declare that the research was conducted in the absence of any commercial or financial relationships that could be construed as a potential conflict of interest.
