# Supplementary material for: Circuits Regulating Pleasure and Happiness: The Evolution of the Amygdalar-Hippocampal-Habenular Connectivity in Vertebrates
Source: Front Neurosci. 2016 Nov 22;10:539. doi: 10.3389/fnins.2016.00539 (PMC5118621; doi:10.3389/fnins.2016.00539)
Supplement: Supplementary file 1 [file Presentation1.PDF]

Supplementary material to figure

Anton J.M. Loonen

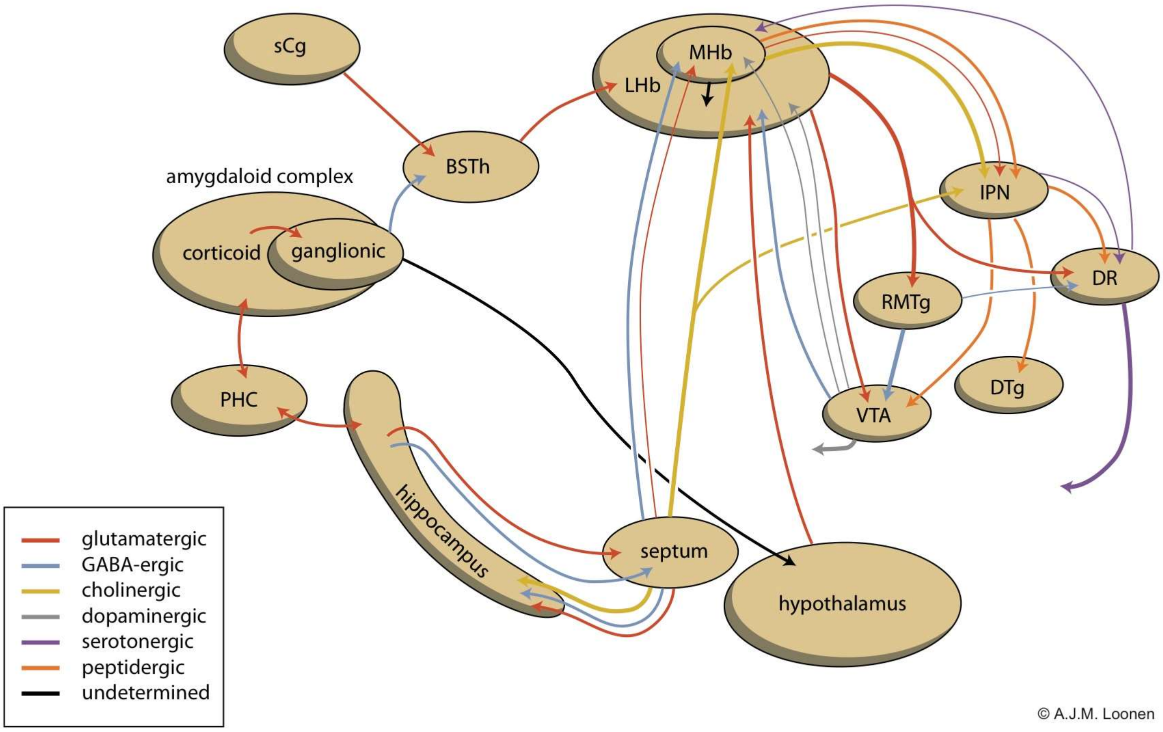


**Figure** Scheme showing the dorsal connectivity of limbic (cortical) system to the midbrain.

BSTh = habenula-projecting part of bed nucleus of the stria terminalis; DR = dorsal raphe nucleus; DTg = dorsal tegmental nucleus; IPN = interpeduncular nucleus; LHb = lateral habenula; MHb = medial habenula; PHC = parahippocampal cortex; RMTg = rostromedial tegmental nucleus; sCg = subgenual cingulate gyrus; VTA = ventral tegmental area.

## *Cerebral cortical areas*

The forerunner of the cerebral cortex (pallium) of the hagfish, which ancestress the lamprey, can (based on their characteristics) better not be divided into medial, dorsal, and lateral pallial fields like those found in gnathostomes. Wicht and Northcutt (1998) have studied the connections of the hagfish pallium and give several reasons for why this pallium is only a homolog of one or two cortical areas of higher developed craniates. In line with this, Medina and Abellán (2009) state that the dorsal pallium does not appear to be present in the lamprey and was likely absent in the first vertebrates. The dorsal pallium gives rise to the majority of the mammalian neocortex (Medina and Abellán, 2009). However, Ocaña et al. (2015) have recently obtained evidence for functional connectivity between the dorsal and dorsomedial part of lateral pallium and certain brainstem motor centers (pretectum, tectum, midbrain tegmentum and locomotor region, and reticolospinal cells) in lampreys. The described brainstem structures may correspond to primate brainstem structures for smooth persuit eye movements (Mustari et al., 2009) and the pedunculopontine nucleus (PPN,

Benarroch, 2013; Gut and Winn, 2016). Both structures are connected with the cerebral neocortex in primates (Mustari et al., 2009; Matsumara et al., 2000; Aravamuthan et al., 2007, 2009). The most likely explanation for the findings of Ocaña et al. (2015) would be, that the part of the lamprey lateral pallium (dorsolateral pallium), hosting the cell bodies of pallio-pretecto-midbrain neurons, develops into the frontal motor cortex in primates. Hence, the lamprey must already have a dorsal pallium that in mammals gives rise to the cerebral neocortex. Therefore, we consider the lamprey to be comparable with the first evolved vertebrate with a dorsal pallium (Loonen and Ivanova, 2015). However, it takes to the transition from primitive sauropsids to mammals before the human neocortex actually starts to develop (Loonen and Ivanova, in preparation). Until amphibians the dorsal pallium can be considered an extension of the medial pallium, which will give rise to part of the human hippocampal complex.

In lampreys the telencephalon consists of paired olfactory bulbs, evaginated hemispheres (lateral pallium) as well as more caudal unevaginated dorsal and medial pallia, each with its own connectivity (Northcutt and Wicht, 1997). Nieuwenhuys and Nicholson (1998) distinguish four areas: the primordium hippocampi (medial pallium), the lobus subhippocampalis (dorsal pallium), the primordium pallii dorsalis (dorsal or general pallium), and the primordium piriforme (lateral pallium). The last two added together form the dorsal and ventral part of the hemisphere (lateral pallium) of Northcutt and Wicht (1997). It has been suggested that only the subhippocampal lobe corresponds to the topological position of the dorsal pallium of gnathostomes (Nieuwenhuys and Nicholson, 1998). However, also the dorsal part of the hemisphere (lateral pallium) may be considered to represent dorsal pallium (Ocaña et al.,2015) and the subhippocampal lobe may also be considered to belong to the medial pallidum. All parts of the pallium receive olfactory input, but input from the dorsal thalamus is largely restricted to hippocampal primordium and the subhippocampal lobe (Nieuwenhuys and Nicholson, 1998; Northcutt and Wicht, 1997). Efferents of the hippocampal primordium run to the brainstem within a so-called hippocampal-pretecto-midbrain bundle (Nieuwenhuys and Nicholson, 1998; Northcutt and Wicht, 1997).

In amphibians (oriental fire-bellied toad) the dorsal pallium has significantly expanded and covers already almost the entire roof of the hemisphere (Roth et al., 2007). Fibres of neurons within these dorsal pallial fields run ipsilaterally to other pallial regions (medial, lateral, ventral), septum, and ventral striatopallidum (Roth et al., 2007). However, projections of the thalamus are certainly not restricted to these new dorsal pallial fields (Roth et al., 2003; Laberge and Roth, 2007; Laberge et al., 2008). Anterior parts of the anuran thalamus are projecting to medial and dorsal pallium, dorsal and ventral striatum, nuclear amygdala, lateral septum and diagonal of Broca nucleus, while visual information is projected to hypothalamus and brainstem (Roth et al., 2003). From electrical recording and anatomical labelling experiments it can be concluded that the anuran dorsal pallium does not yet has achieved its human input analyzing and output generating role (Roth et al., 2003; Laberge and Roth, 2007; Laberge et al., 2008), but is still part of a more extensive 'limbic' behavioral control system including almost all pallial and subpallial regions.

In more recent jawed vertebrates the input to the dorsal thalamus largely increases and this leads to a significant expansion of the dorsal pallium (Butler, 1994a, 1994b). The dorsal thalamus, which serves as an input channel to the cerebral cortex, consists of two divisions called lemnothalamus and collothalamus. In mammals both divisions with the corresponding lateral dorsal pallial field largely expanded (Butler, 1994b). The medial part of the lemnothalamic medial division forms the subicular, cingulate, prefrontal, sensorimotor, and related cortices in mammals. The lateral part forms striate (visual) cortex. Specific fields within the collothalamic lateral division of the dorsal pallium form the extrastriate visual, auditory, secondary somatosensory, and related cortices in mammals (Butler,

1994b). The described expansion resulted probably in a total displacement of ventral and medial pallial fields. It is tempting to speculate that the primordium hippocampi (medial pallium) and primordium piriforme (ventrolateral pallium) of lamprey give rise to the human hippocampal complex and the most caudal edge of the frontal lobe (including olfactory tubercle) and cortical regions of the amygdaloid complex in the temporal lobe.

The amygdaloid complex is a heterogeneous group of 13 nuclei and cortical areas located in the medial temporal lobe just rostral to the hippocampal formation (Freese and Amaral, 2009). The complex can neuroanatomically be divided into 'deep nuclei', 'superficial nuclei' and 'remaining nuclei' (Freese and Amaral, 2009). Both the cortical amygdalar nuclei and the basolateral amygdalar nuclear complex, which is located deep to it, have cortex-like cell types (McDonald and Mott, 2016). In contrast, the so called 'extended amygdalar nuclei' contain predominantly GABA-ergic spiny projection neurons, like the striatum (McDonald and Mott, 2016).

Three or four components of amygdaloid connectivity can be distinguished (Swanson and Petrovich, 1998; Price, 2003): the accessory olfactory division,  the main olfactory division, the autonomic division and the frontotemporal division. The first two may be added together. The frontotemporal division is often primarily associated with strong bidirectional interactions with the prefrontal cortex and hippocampal formation (Benarroch, 2015a; Pitkänen, 2000), but the amygdalohippocampal system can also be considered to be an output channel of the amygdaloid complex. The connectivity of the deep corticoid amygdaloid complex from and to the hippocampal complex is mediated through parahippocampal regions, including pre-, and parasubiculum, the entorhinal as well as the perirhinal cortices and the postrhinal (in non-primate mammalians), or parahippocampal cortex (in primates) (Witter, 2002). The last three regions are heavily targeted by olfactory endopiriform cortex, claustrum, basolateral amygdala, medial septum and dorsal midline thalamic nuclei (Pereira et al., 2016). Output is primarily provided to the olfactory piriform transition area, basolateral amygdala, claustrum and dorsal medial thalamic nuclei (Agster et al., 2016). Via the fornix the hippocampus sends a GABAergic connection to the medial septum and a glutamatergic connection to the lateral septum (Khakpai et al., 2013; Nieuwenhuys et al., 2008). Reciprocally, cholinergic and to a far less extent GABAergic and glutamatergic fibres coming from the medial septum-diagonal band of Broca complex run through the fornix to the hippocampus (Khakpai et al., 2013; Nieuwenhuys et al., 2008).

*Basal ganglia*

Loonen and Ivanova (2015) have hypothesized that the lampreys striatum went op into the centromedial amygdala. The centromedial nucleus is a ganglionic structure with a primary unidirectional and topographically organized flow of information (Benarroch, 2015a). The central nucleus receives moderate to heavy inputs from the lateral, basal, accessory basal, medial and anterior cortical nuclei, next to several extra-amygdaloid structures (Pitkänen, 2000). It provides substantial output to the bed nucleus of stria terminalis (amygdaloid pallidum), many hypothalamic nuclei and several nuclei in the midbrain, pons, and medulla. The medial amygdala receives many projections from the lateral, accessory basal, posterior cortical nuclei, and amygdalo-hippocampal area. Lighter projections originate in the basal nucleus, anterior cortical nucleus and periamygdaloid cortex. Extra-amygdaloid projections originate within the prefrontal cortex, the bed nucleus of the stria terminalis and the hypothalamus. Output is mainly provided to the olfactory system, bed nucleus of the stria terminalis (amygdaloid pallidum) and thalamus (Pitkänen, 2000). The basolateral complex is connected with many neocortical areas and vice versa with the medial temporal lobe

memory system (Benarroch, 2015a; Pitkänen, 2000). The connectivity from and to the hippocampal complex is mediated through parahippocampal regions, including pre-, and parasubiculum, the entorhinal as well as the perirhinal cortices and the postrhinal (in non-primate mammalians), or parahippocampal cortex (in primates) (Witter, 2002). The last three regions are heavily targeted by olfactory endopiriform cortex, claustrum, basolateral amygdala, medial septum and dorsal midline thalamic nuclei (Pereira et al., 2016). Output is primarily provided to the olfactory piriform transition area, basolateral amygdala, claustrum and dorsal medial thalamic nuclei (Agster et al., 2016). The septohippocampal system can be considered as the neural substrate regulating the behavioral inhibition system that modulates reaction to stimuli indicating adverse events (Gray, 1982; Hahn et al., 2010). It has been demonstrated anticipation of monetary loss elicited activation in the hippocampus as well as in the amygdala (Hahn et al., 2010). Via the fornix the hippocampus sends a GABAergic connection to the medial septum and a glutamatergic connection to the lateral septum (Khakpai et al., 2013; Nieuwenhuys et al., 2008). Reciprocally, cholinergic and to a far less extent GABAergic and glutamatergic fibres coming from the medial septum-diagonal band of Broca complex run through the fornix to the hippocampus (Khakpai et al., 2013; Nieuwenhuys et al., 2008).

*Limbic basal ganglia*

The position of the habenula-projecting globus pallidus (GPh) within this figure can be subject of debate. The lamprey's GPh receives excitatory glutamatergic input from the lateral pallium, as well as inhibitory GABAergic input from the striosomes homologue of the striatum (Stephenson-Jones et al., 2013). It has been suggested that the GPh has been conserved as the border region of the globus pallidus (GPb) (Stephenson-Jones et al., 2013). These GPb cells probably correspond to lateral habenula (LHb)-projecting excitatory neurons, which respond to reward or no-reward indicators (Hong and Hikosaka, 2008). They confirmed the results of Parent et al. (2001), who distinguished two types of projection neurons in the border of the internal pallidum of primates, one acting upon thalamic and brainstem premotor neurons, whereas the other type upon LHb neurons. In the rat these neurons projecting from the entopeduncular neurons (EPN, equivalent to globus pallidus) to the habenula are targeted by striosome neurons from the neostriatum (Rajakumar et al., 1993). The role of LHb-projecting GPb neurons was confirmed recently in primates, but these authors also found that LHb-projecting neurons originated within the ventral pallidum (VP) (Hong and Hikosaka, 2013). The ventral pallidum (VP) receives most input from the nucleus accumbens (ventral striatum) (Groenewegen, 2007) and projects to the LHb (Haber et al., 1993). However, the majority of descending efferent projection from the ventral pallidum in monkeys terminates primarily in the subthalamic nucleus and adjacent lateral hypothalamus, in the substantia nigra, next to the lateral habenular nucleus. Ventral striatum and ventral pallidum are part of the cortical-striato-pallido-(hypo)thalamo-cortical circuit regulating motivation to reward-seeking (Nucleus Accumbens Core; NAcbC) and misery-fleeing behavior (Nucleus Accumbens Shell; NAcbS). Dorsal striatum and global pallidus are primary involved in decision-making and control of voluntary motions (Grillner and Robertson, 2015). The process of decision-making is dysfunctional in many psychic disorders (e.g., bipolar disorders and anxiety disorders like obsessive-compulsive disorder). The LHb plays an essential role in regulating the activity of the extrapyramidal circuits by modifying input from dopaminergic input from the midbrain to the striatum. Probably, GPb neurons supply the LHb with feed-back information to adapt its activity when necessary.

An important discovery during studying the embryological development of anuran basal ganglia was the finding that the bed nucleus of the stria terminalis (BST) and part of the septum are also of pallidal instead of striatal origin (González et al., 2014; Moreno et al., 2012). This is interesting

because the BST is a suitable structure to execute the limbic component of lamprey habenula-projecting globus pallidus. This finding made clear that the entire lamprey striatopallidal system went up into extended amygdala as described in the original concept of Heimer and Van Hoesen (2006) so including the centromedial amygdala and bed nucleus of the stria terminalis. The architecture and connectivity of the rat BST has been studied in detail by Larry Swanson and collaborators (Dong and Swanson, 2003, 2004a, 2004b, 2006a, 2006b, 2006c; Dong et al., 2000, 2001a; Ju and Swanson, 1989; Ju et al., 1989). It becomes evident that the BST is an extremely complex set of nuclei, which can be separated into dorsal, lateral and ventral areas (Ju and Swanson, 1989). These nuclei receive input from the central amygdaloid nucleus (innervating various parts of the anterior BST division) and medial amygdaloid nucleus (preferentially innervating the posterior BST division), but not from the superficial and deep corticoid nuclei of the amygdala (Dong et al., 2001b). It is concluded that BST is a rostral differentiation of the pallidum receiving massive GABAergic input from centromedial amygdala and giving again GABAergic output to brainstem motor systems and thalamocortical re-entrant loops (Dong et al., 2001b). Moreover, dense peptidergic transmission from and to BST became evident when studying its chemoarchitecture (Ju et al., 1989). The organization of the projections from and to the separate BST nuclei is too complex to be described within the context of this overview. Viewed broadly, BST posterior division cell groups share massive bidirectional connections with the medial amygdaloid nucleus and other amygdaloid components of the accessory olfactory system and they send massive projections to hypothalamic control centres regulating reproduction and defence (Dong and Swanson, 2006a). The BST anterolateral group projects to the ventral autonomic control network, to midbrain structures modulating the expression of orofacial and locomotor somatosensory responses, and to the ventral striatopallidal system. This suggests that the anterolateral group is primary involved in appetitive feeding (eating and drinking) behaviour (Dong and Swanson, 2006a). The lateral habenula hardly receives any fibres from these BST areas. However, the anteromedial area (BSTamg) and even more extensively the dorsomedial nucleus (BSTdm) of anteromedial BST division projects to medial, resp. caudal regions of the lateral habenula (Dong and Swanson, 2006a, 2006c). Lateral habenula afferents from the BST are also described by Felton et al. (1999), but these authors give any specification. The neurochemical characteristics of these connections have not been elucidated. In our opinion, it is very well possible that the anteromedial division of the rat BST contains glutamatergic neurons which are running to the lateral habenula and have similar function as lamprey GPh neurons to inhibit the activity of dopaminergic midbrain nuclei which results in inhibition of behaviour when its positive results are disappointing (comparable to anti-reward sensing). Moreover, the anterior BST division receives input from hippocampus (ventral subiculum) and infralimbic cortex (comparable with the human subgenual anterior cingulate cortex, Brodmann Area 25, BA25). It should be kept in mind that the ventral subiculum projects substantially to the infralimbic area (Dong et al., 2001b). This connectivity probably corresponds to the cortical input to the habenula-projecting globus pallidus.

Hence, the GPh of our earliest vertebrate ancestors corresponds to the border region of the globus pallidus (GPb) within the dorsal extrapyramidal system, with a subset of neurons of the ventral pallidum (VPh) within the ventral extrapyramidal system and with a subset of neurons of the bed nucleus of the stria terminalis (BSTh) within the amygdaloid system.

### *Epithalamus and brainstem*

The stria medullaris is the main habenular input and the fasciculus retroflexus is the primary output of the habenula (Benarroch, 2015b; Bianco and Wilson, 2009; Klemm, 2004; Sutherland, 1982). The habenula receives signals from the septum, hippocampus, ventral pallidum, lateral hypothalamus,

global pallidus, and other areas of basal ganglia. The septum, particularly the medial septum and the adjacent nucleus of the diagonal band of Broca, is the main input to the MHb, whereas the remaining regions project mainly to the LHb (Benarroch, 2015b; Klemm 2004). The input to the MHb from these septal areas is mainly cholinergic and gamma-aminobutyric acid (GABA)ergic, although some inputs are glutamatergic (Benarroch, 2015b). Moreover, the MHb receives dopaminergic input from the VTA and adrenergic (norepinephrine) input from the locus coeruleus (Benarroch, 2015b; Bianco and Wilson, 2009). The LHb primarily receives glutamatergic afferents from the preoptic area, lateral hypothalamus, the entopeduncular nucleus (EPN; analog of globus pallidus in primates) and from anterior cingulate and the medial prefrontal cortex (Benarroch, 2015b). Moreover, the LHB also receives strong GABAergic innervations (Poller et al., 2013) from various brain regions, including e.g. the EPN, VTA, and nucleus accumbens. However, most of their functional inputs are still unknown (Shabel et al., 2012). Hence, there is a large heterogeneity in GABAergic inputs onto LHb neurons. Although the output of the basal ganglia is thought to be primarily inhibitory, Shabel et al. (2012) have shown that transmission from the basal ganglia to the LHb is excitatory, glutamatergic, and suppressed by serotonin. This probably corresponds to input from the GPb. The LHb also receives dopaminergic innervation from the VTA, serotonergic innervation from the medial raphe nucleus, and adrenergic input from the locus coeruleus, next to an unique population of inhibitory VTA neurons that synthesize both DA and GABA (Benarroch, 2015b; Stamatakis et al., 2012, 2013). Although the MHb is connected to the LHb, there is no connection from LHb to the Mhb (Kim and Chang, 2005).

Information encoded by the LHb and the MHb is transmitted through the fasciculus retroflexus (FR) axon bundle to midbrain monoaminergic nuclei, such as the dopaminergic ventral tegmental area (VTA) and substantia nigra pars compacta, and the serotonergic raphe nuclei (Hikosaka, 2010). The FR is divided into two regions. The outer region originates in the LHb and projects mainly to the rostromedial tegmental nucleus (RMTg), next to numerous monoaminergic nuclei in the mid- and hindbrain (Bianco and Wilson, 2009). These efferents are predominantly glutamatergic, as well as some GABAergic and cholinergic (Bianco and Wilson, 2009). The RMTg, which is also named as the “tail” of the VTA, is a small nucleus that contains mainly inhibitory GABAergic cells and thereby regulates activity of VTA/substantia nigra compacta (SNc) and the dorsal raphe nucleus (Benarroch, 2015b). More specifically, LHb neurons predominantly inhibit dopaminergic (DA) neurons of the midbrain (Ji and Shepard, 2007; Matsumoto and Hikosaka, 2007). So, electrical stimulation of LHb abolished the firing of ~ 90% of DA neurons of VTA and SNc (Christoph et al., 1986; Matsumoto and Hikosaka, 2007). Vice versa, lesion of LHb increases DA turnover in terminal projection areas of these midbrain nuclei (Lecourtier et al., 2008). Similarly, through its RMTg projection stimulation of the LHb causes transient inhibition of the firing activity of serotonergic neurons in the raphe nucleus (Wang and Aghajanian,1977). LHb neurons also directly targets the dopaminergic VTA (Lammel et al 2012) and substantia nigra pars compacta, the serotonergic median and dorsal raphe nucleus (DRN), cholinergic laterodorsal tegmentum and noradrenergic locus coeruleus (Herkenham and Nauta, 1979). There is also a feedback from VTA and the raphe nuclei to the LHb (Herkenham and Nauta, 1977; Gruber et al 2007). The LHb neurons express tyrosine hydroxylase (TH) and DA type 2 and 4 receptors (Geisler et al 2003; Gruber et al 2007; Aizawa et al 2012; Good et al 2013). A single-pulse stimulation of the VTA, likely activating both glutamatergic and GABAergic projections, inhibits the firing of ~ 90% of the LHb neurons, whereas tetanic stimulation increases the activity of LHb units (Shen et al 2012).

The inner area of the FR originates in the MHb and projects to the interpeduncular nucleus (IPN; Bianco & Wilson, 2009; Benarroch, 2015b; Klemm, 2004; Sutherland, 1982). The MHb contains both cholinergic neurons (in its ventral two-thirds) and dorsally located substance P-containing neurons,

which innervate the ventral & dorsal vs. the lateral IPN, respectively (Artymyshyn and Murray, 1985; Contestabile et al 1987). This neuronal pathway is highly conserved across various species (Broms et al., 2015). The results of Qin and Luo (2009) suggest that, at least in mice, also glutamate is used as a transmitter next to acetylcholine and substance P. The MHb is also the main source of the input of the IPN (Bianco & Wilson, 2009; Klemm, 2004; Morley, 1986), although cholinergic fibres may also originate in the posterior septum (Contestabile and Fonnum, 1983; Fonnum and Contestaile, 1984). The IPN is a singular, unpaired structure located at the ventral midline of the midbrain (Klemm, 2004; Morley, 1986). The major efferent pathways originating in the IPN project to the dorsal tegmental nucleus (Morley, 1986), the VTA (Klemm, 2004) and the raphe nuclei (Bianco & Wilson, 2009; Klemm, 2004). However, the IPN is well known for its widespread projections both ascending and descending (Klemm, 2004; Morley, 1986). Apart from a low number of serotonergic neurons (continuous with the B8 cell group of the median raphe nucleus) numerous peptidergic neurons (substance P, met-enkephalin, somatostatin) have been identified within the IPN (Morley, 1986).

Neurons of LHb are morphologically heterogeneous projecting cells with long-range axons and small dendritic arborizations (Weiss and Veh, 2011). Most of the LHb neurons are glutamatergic with enriched expression of vesicular glutamate transporter (VGluT2) (Li et al., 2011; Stamatakis and Stuber, 2012). Also, there is a small population of inhibitory GABAergic interneurons (Smith et al., 1987).

The MHb is distinctive from the LHb in that it co-expresses the mRNA for all three of α3, α5, and β4 nAChRs subunits at very high (α3 and β4) or moderate (α5) levels (Bierut, 2011). IPN, the output from the MHb, is also enriched in α5 and β4 nAChR subunits expression. Also muscarinic cholinergic receptors have been identified within the INP (Kuhar et al., 1975), though less extensive distributed than nAChRs (Schwartz, 1986; Rotter et al, 1979). Spencer et al. (1986) demonstrated that within the IPN muscarinic receptors were almost exclusively M2-class (inhibitory) autoreceptors on cholinergic terminals.

References

Agster KL, Tomás Pereira I, Saddoris MP, Burwell RD. Subcortical connections of the perirhinal, postrhinal, and entorhinal cortices of the rat. II. efferents. Hippocampus. 2016 Apr 22. doi: 10.1002/hipo.22600. [Epub ahead of print]

Aizawa H, Kobayashi M, Tanaka S, Fukai T, Okamoto H. Molecular characterization of the subnuclei in rat habenula. J Comp Neurol. 2012;520(18):4051-66.

Aravamuthan BR, Muthusamy KA, Stein JF, Aziz TZ, Johansen-Berg H. Topography of cortical and subcortical connections of the human pedunculopontine and subthalamic nuclei. Neuroimage. 2007;37(3):694-705.

Aravamuthan BR, McNab JA, Miller KL, Rushworth M, Jenkinson N, Stein JF, Aziz TZ. Cortical and subcortical connections within the pedunculopontine nucleus of the primate Macaca mulatta determined using probabilistic diffusion tractography. J Clin Neurosci. 2009;16(3):413-20.

Artymyshyn R, Murray M. Substance P in the interpeduncular nucleus of the rat: normal distribution and the effects of deafferentation. J Comp Neurol. 1985;231(1):78-90.

Benarroch EE. Pedunculopontine nucleus: functional organization and clinical implications. Neurology. 2013;80(12):1148-55

Benarroch EE. The amygdala: functional organization and involvement in neurologic disorders. Neurology. 2015a;84(3):313-24.

Benarroch EE. Habenula: Recently recognized functions and potential clinical relevance. Neurology 2015b;85(11):992-1000.

Bianco IH, Wilson SW. The habenular nuclei: a conserved asymmetric relay station in the verte-brate brain. Philos Trans R Soc Lond B Biol Sci 2009;364(1519):1005-20.

Bierut LJ. Genetic vulnerability and susceptibility to substance dependence. Neuron. 2011;69(4):618-27.

Broms J, Antolin-Fontes B, Tingström A, Ibañez-Tallon I. Conserved expression of the GPR151 receptor in habenular axonal projections of vertebrates. J Comp Neurol. 2015;523(3):359-80.

Butler AB. The evolution of the dorsal thalamus of jawed vertebrates, including mammals: cladistic analysis and a new hypothesis. Brain Res Brain Res Rev. 1994a;19(1):29-65.

Butler AB. The evolution of the dorsal pallium in the telencephalon of amniotes: cladistic analysis and a new hypothesis. Brain Res Brain Res Rev. 1994b;19(1):66-101.

Christoph GR, Leonzio RJ, Wilcox KS. Stimulation of the lateral habenula inhibits dopamine-containing neurons in the substantia nigra and ventral tegmental area of the rat. J Neurosci. 1986;6(3):613-9.

Contestabile A, Fonnum F. Cholinergic and GABAergic forebrain projections to the habenula and nucleus interpeduncularis: surgical and kainic acid lesions. Brain Res 1983;275(2):287-97.

Contestabile A, Villani L, Fasolo A, Franzoni MF, Gribaudo L, Oktedalen O, Fonnum F. Topography of cholinergic and substance P pathways in the habenulo-interpeduncular system of the rat. An immunocytochemical and microchemical approach. Neuroscience. 1987;21(1):253-70.

Dong HW, Swanson LW. Projections from the rhomboid nucleus of the bed nuclei of the stria terminalis: implications for cerebral hemisphere regulation of ingestive behaviors. J Comp Neurol. 2003;463(4):434-72.

Dong HW, Swanson LW. Organization of axonal projections from the anterolateral area of the bed nuclei of the stria terminalis. J Comp Neurol. 2004a;468(2):277-98.

Dong HW, Swanson LW. Projections from bed nuclei of the stria terminalis, posterior division: implications for cerebral hemisphere regulation of defensive and reproductive behaviors. J Comp Neurol. 2004b;471(4):396-433.

Dong HW, Swanson LW. Projections from bed nuclei of the stria terminalis, dorsomedial nucleus: implications for cerebral hemisphere integration of neuroendocrine, autonomic, and drinking responses. J Comp Neurol. 2006a;494(1):75-107.

Dong HW, Swanson LW. Projections from bed nuclei of the stria terminalis, magnocellular nucleus: implications for cerebral hemisphere regulation of micturition, defecation, and penile erection. J Comp Neurol. 2006b;494(1):108-41.

Dong HW, Swanson LW. Projections from bed nuclei of the stria terminalis, anteromedial area: cerebral hemisphere integration of neuroendocrine, autonomic, and behavioral aspects of energy balance. J Comp Neurol. 2006c;494(1):142-78.

Dong H, Petrovich GD, Swanson LW. Organization of projections from the juxtacapsular nucleus of the BST: a PHAL study in the rat. Brain Res. 2000;859(1):1-14.

Dong HW, Petrovich GD, Watts AG, Swanson LW. Basic organization of projections from the oval and fusiform nuclei of the bed nuclei of the stria terminalis in adult rat brain. J Comp Neurol. 2001a;436(4):430-55.

Dong HW, Petrovich GD, Swanson LW. Topography of projections from amygdala to bed nuclei of the stria terminalis. Brain Res Brain Res Rev. 2001b;38(1-2):192-246.

Felton TM, Linton L, Rosenblatt JS, Morell JI. First and second order maternal behavior related afferents of the lateral habenula. Neuroreport. 1999;10(4):883-7.

Fonnum F, Contestabile A. Colchicine neurotoxicity demonstrates the cholinergic projection from the supracommissural septum to the habenula and the nucleus interpeduncularis in the rat. J Neurochem. 1984;43(3):881-4.

Freese JL, Amaral DG. Neuroanatomy of the primate amygdala. In: Whalen PJ, Phelps EA, eds. The human amygdala. New York: The Guilford Press, 2009; 3-42.

Geisler S, Andres KH, Veh RW. Morphologic and cytochemical criteria for the identification and delineation of individual subnuclei within the lateral habenular complex of the rat. J Comp Neurol. 2003;458(1):78-97.

González A, Morona R, Moreno N, Bandín S, López JM. Identification of striatal and pallidal regions in the subpallium of anamniotes. Brain Behav Evol. 2014;83(2):93-103.

Good CH, Wang H, Chen YH, Mejias-Aponte CA, Hoffman AF, Lupica CR. Dopamine D4 receptor excitation of lateral habenula neurons via multiple cellular mechanisms. J Neurosci. 2013;33(43):16853-64.

Gray JA. The neuropsychology of anxiety: an enquiry into the function of the septo-hippocampal system. Oxford, UK: Oxford University Press, 1982.

Groenewegen HJ. The ventral striatum as an interface between the limbic and motor systems. CNS Spectr 2007;12(12):887-92.

Gruber C, Kahl A, Lebenheim L, Kowski A, Dittgen A, Veh RW. Dopaminergic projections from the VTA substantially contribute to the mesohabenular pathway in the rat. Neurosci Lett. 2007;427(3):165-70.

Gut NK, Winn P. The pedunculopontine tegmental nucleus-A functional hypothesis from the comparative literature. Mov Disord. 2016 Feb 16. doi: 10.1002/mds.26556.

Haber SN, Lynd-Balta E, Mitchell SJ. The organization of the descending ventral pallidal projections in the monkey. J Comp Neurol. 1993;329(1):111-28.

Hahn T, Dresler T, Plichta MM, Ehlis AC, Ernst LH, Markulin F, Polak T, Blaimer M, Deckert J, Lesch KP, Jakob PM, Fallgatter AJ. Functional amygdala-hippocampus connectivity during anticipation of aversive events is associated with Gray's trait "sensitivity to punishment". Biol Psychiatry. 2010;68(5):459-64.

Heimer L, Van Hoesen GW. The limbic lobe and its output channels: implications for emotional functions and adaptive behavior. Neurosci Biobehav Rev 2006;30(2):126-47.

Herkenham M, Nauta WJ. Afferent connections of the habenular nuclei in the rat. A horseradish peroxidase study, with a note on the fiber-of-passage problem. J Comp Neurol. 1977;173(1):123-46.

Herkenham M, Nauta WJ. Efferent connections of the habenular nuclei in the rat. J Comp Neurol. 1979;187(1):19-47.

Hong S, Hikosaka O. The globus pallidus sends reward-related signals to the lateral habenula. Neuron. 2008;60(4):720-9.

Ji H, Shepard PD. Lateral habenula stimulation inhibits rat midbrain dopamine neurons through a GABA(A) receptor-mediated mechanism. J Neurosci. 2007;27(26):6923-30.

Ju G, Swanson LW. Studies on the cellular architecture of the bed nuclei of the stria terminalis in the rat: I. Cytoarchitecture. J Comp Neurol. 1989;280(4):587-602.

Ju G, Swanson LW, Simerly RB. Studies on the cellular architecture of the bed nuclei of the stria terminalis in the rat: II. Chemoarchitecture. J Comp Neurol. 1989;280(4):603-21.

Khakpai F, Nasehi M, Haeri-Rohani A, Eidi A, Zarrindast MR. Septo-hippocampo-septal loop and memory formation. Basic Clin Neurosci. 2013;4(1):5-23.

Kim U, Chang SY. Dendritic morphology, local circuitry, and intrinsic electrophysiology of neurons in the rat medial and lateral habenular nuclei of the epithalamus. J Comp Neurol. 2005;483(2):236-50.

Klemm WR. Habenular and interpeduncularis nuclei: shared components in multiple-function networks. Med Sci Monit. 2004;10(11):RA261-73.

Kowski AB, Veh RW, Weiss T. Dopaminergic activation excites rat lateral habenular neurons in vivo. Neuroscience. 2009;161(4):1154-65.

Kuhar MJ, DeHaven RN, Yamamura HI, Rommel-Spacher H, Simon JR. Further evidence for cholinergic habenulo-interpeduncular neurons: pharmacologic and functional characteristics. Brain Res 1975;97(2):265-75.

Laberge F, Roth G. Organization of the sensory input to the telencephalon in the fire-bellied toad, Bombina orientalis. J Comp Neurol. 2007;502(1):55-74.

Laberge F, Mühlenbrock-Lenter S, Dicke U, Roth G. Thalamo-telencephalic pathways in the fire-bellied toad Bombina orientalis. J Comp Neurol. 2008;508(5):806-23.

Lammel S, Lim BK, Ran C, Huang KW, Betley MJ, Tye KM, Deisseroth K, Malenka RC. Input-specific control of reward and aversion in the ventral tegmental area. Nature. 2012;491(7423):212-7.

Lecourtier L, Defrancesco A, Moghaddam B. Differential tonic influence of lateral habenula on prefrontal cortex and nucleus accumbens dopamine release. Eur J Neurosci. 2008;27(7):1755-62.

Li B, Piriz J, Mirrione M, Chung C, Proulx CD, Schulz D, Henn F, Malinow R. Synaptic potentiation onto habenula neurons in the learned helplessness model of depression. Nature. 2011;470(7335):535-9.

Liang F, Hatanaka Y, Saito H, Yamamori T, Hashikawa T. Differential expression of gamma-aminobutyric acid type B receptor-1a and -1b mRNA variants in GABA and non-GABAergic neurons of the rat brain. J Comp Neurol. 2000;416(4):475-95.

Loonen AJ, Ivanova SA. Circuits regulating pleasure and happiness: the evolution of reward-seeking and misery-fleeing behavioral mechanisms in vertebrates. Front Neurosci 2015;9:394.

Loonen AJ, Ivanova SA. Circuits regulating pleasure and happiness: the evolution of the amygdalar-hippocampal-habenular connection in vertebrates. In preparation.

Maroteaux M, Mameli M. Cocaine evokes projection-specific synaptic plasticity of lateral habenula neurons. J Neurosci. 2012;32(36):12641-6.

Matsumoto M, Hikosaka O. Lateral habenula as a source of negative reward signals in dopamine neurons. Nature. 2007;447(7148):1111-5.

Matsumura M, Nambu A, Yamaji Y, Watanabe K, Imai H, Inase M, Tokuno H, Takada M. Organization of somatic motor inputs from the frontal lobe to the pedunculopontine tegmental nucleus in the macaque monkey. Neuroscience. 2000;98(1):97-110.

McDonald AJ, Mott DD. Functional neuroanatomy of amygdalohippocampal interconnections and their role in learning and memory. J Neurosci Res. 2016. doi: 10.1002/jnr.23709. [Epub ahead of print].

Moreno, N., Morona, R., López, J.M., Domínguez, L., Joven, A., Bandín, S., and González, A. (2012). Characterization of the bed nucleus of the stria terminalis in the forebrain of anuran amphibians. J. Comp. Neurol. 520, 330-363.

Morley BJ. The interpeduncular nucleus. Int Rev Neurobiol 1986;28:157-82.

Mustari MJ, Ono S, Das VE. Signal processing and distribution in cortical-brainstem pathways for smooth pursuit eye movements. Ann N Y Acad Sci. 2009;1164:147-54

Nieuwenhuys R, Nicholson C. Chapter 10: Lampreys, Petromyzontoidea. In: Nieuwenhuys R, Ten Donkelaar HJ, Nicholson C, eds. The central nervous system of vertebrates. Berlin, Heidelberg: Springer-Verlag, 1998:397-495.

Nieuwenhuys R, Voogd J, Van Huijzen C. 12 Telencephalon: hippocampus and related structures. In: The human central nervous system, 4$^{th}$ edition. Berlin, Heidelberg: Springer, 2008; 361-400.

Parent M, Lévesque M, Parent A. Two types of projection neurons in the internal pallidum of primates: single-axon tracing and three-dimensional reconstruction. J Comp Neurol 2001;439(2):162-75.

Pereira IT, Agster KL, Burwell RD. Subcortical connections of the perirhinal, postrhinal, and entorhinal cortices of the rat. I. afferents. Hippocampus. 2016 Apr 27. doi: 10.1002/hipo.22603. [Epub ahead of print]

Pitkänen A. Connectivity of the rat amygdaloid complex. In: JP Aggleton, ed. The amygdala - a functional analysis, second edition. New York: Oxford University Press, 2000; 31-115.

Poller WC, Madai VI, Bernard R, Laube G, Veh RW. A glutamatergic projection from the lateral hypothalamus targets VTA-projecting neurons in the lateral habenula of the rat. Brain Res. 2013;1507:45-60.

Price JL. Comparative aspects of amygdala connectivity. Ann N Y Acad Sci. 2003;985:50-8.

Qin C, Luo M. Neurochemical phenotypes of the afferent and efferent projections of the mouse medial habenula. Neuroscience 2009;161(3):827-37.

Rajakumar N, Elisevich K, Flumerfelt BA. Compartmental origin of the striato-entopeduncular projection in the rat. J Comp Neurol 1993;331(2):286-96.

Robertson B, Kardamakis A, Capantini L, Pérez-Fernández J, Suryanarayana SM, Wallén P, Stephenson-Jones M, Grillner S. The lamprey blueprint of the mammalian nervous system. Prog Brain Res 2014;212:337-49.

Rodríguez-Pallares J, Caruncho HJ, López-Real A, Wójcik S, Guerra MJ, Labandeira-García JL. Rat brain cholinergic, dopaminergic, noradrenergic and serotonergic neurons express GABAA receptors derived from the alpha3 subunit. Receptors Channels. 2001;7(6):471-8.

Roth G, Grunwald W, Dicke U. Morphology, axonal projection pattern, and responses to optic nerve stimulation of thalamic neurons in the fire-bellied toad Bombina orientalis. J Comp Neurol. 2003;461(1):91-110.

Roth G, Laberge F, Mühlenbrock-Lenter S, Grunwald W. Organization of the pallium in the fire-bellied toad Bombina orientalis. I: Morphology and axonal projection pattern of neurons revealed by intracellular biocytin labeling. J Comp Neurol. 2007;501(3):443-64.

Rotter A, Birdsall NJ, Field PM, Raisman G. Muscarinic receptors in the central nervous system of the rat. II. Distribution of binding of [3H]propylbenzilylcholine mustard in the midbrain and hindbrain. Brain Res 1979;180(2):167-83.

Schwartz RD. Autoradiographic distribution of high affinity muscarinic and nicotinic cholinergic receptors labeled with [3H]acetylcholine in rat brain. Life Sci 1986;38(23):2111-9.

Shabel SJ, Proulx CD, Trias A, Murphy RT, Malinow R. Input to the lateral habenula from the basal ganglia is excitatory, aversive, and suppressed by serotonin. Neuron. 2012;74(3):475-81.

Shen X, Ruan X, Zhao H. Stimulation of midbrain dopaminergic structures modifies firing rates of rat lateral habenula neurons. PLoS One. 2012;7(4):e34323.

Smith Y, Séguéla P, Parent A. Distribution of GABA-immunoreactive neurons in the thalamus of the squirrel monkey (Saimiri sciureus). Neuroscience. 1987;22(2):579-91.

Spencer DG Jr, Horváth E, Traber J. Direct autoradiographic determination of M1 and M2 musca-rinic acetylcholine receptor distribution in the rat brain: relation to cholinergic nuclei and projections. Brain Res. 1986;380(1):59-68.

Stamatakis AM, Stuber GD. Activation of lateral habenula inputs to the ventral midbrain promotes behavioral avoidance. Nat Neurosci. 2012;15(8):1105-7.

Stamatakis AM, Jennings JH, Ung RL, Blair GA, Weinberg RJ, Neve RL, Boyce F, Mattis J, Ra-makrishnan C, Deisseroth K, Stuber GD. A unique population of ventral tegmental area neurons inhibits the lateral habenula to promote reward. Neuron. 2013;80(4):1039-53.

Stephenson-Jones M, Kardamakis AA, Robertson B, Grillner S. Independent circuits in the basal ganglia for the evaluation and selection of actions. Proc Natl Acad Sci U S A 2013;110(38):E3670-9.

Swanson LW, Petrovich GD. What is the amygdala? Trends Neurosci. 1998;21(8):323-31.

Sutherland RJ. The dorsal diencephalic conduction system: a review of the anatomy and functions of the habenular complex. Neurosci Biobehav Rev. 1982;6(1):1-13.

Wang RY, Aghajanian GK. Physiological evidence for habenula as major link between forebrain and midbrain raphe. Science. 1977;197(4298):89-91.

Wicht H, Northcutt RG. Telencephalic connections in the Pacific hagfish (Eptatretus stouti), with special reference to the thalamopallial system. J Comp Neurol. 1998;395(2):245-60.

Weiss T, Veh RW. Morphological and electrophysiological characteristics of neurons within identi-fied subnuclei of the lateral habenula in rat brain slices. Neuroscience. 2011;172:74-93.

Witter M. Chapter one: The parahippocampal region: past, present, and future. In: M Witter, F. Wouterlood, eds. The parahippocampal region: organization and role in cognitive function. Oxford, UK: Oxford University Press, 2002; 3-19.
